# Phosphatidylserine-deficient small extracellular vesicle is a major somatic cell-derived sEV subpopulation in blood

**DOI:** 10.1016/j.isci.2021.102839

**Published:** 2021-07-10

**Authors:** Akihiro Matsumoto, Yuki Takahashi, Kosuke Ogata, Shimpei Kitamura, Naoki Nakagawa, Aki Yamamoto, Yasushi Ishihama, Yoshinobu Takakura

**Affiliations:** 1 Department of Biopharmaceutics and Drug Metabolism, Graduate School of Pharmaceutical Sciences, Kyoto University, Sakyo-ku, Kyoto 606-8501, Japan; 2 Department of Molecular and Cellular BioAnalysis, Graduate School of Pharmaceutical Sciences, Kyoto University, Kyoto 606-8501, Japan

**Keywords:** Molecular physiology, Cell biology, Proteomics

## Abstract

Small extracellular vesicles (sEVs) are important mediators of intercellular communication with respect to diverse pathophysiological processes. Here, we determined novel phosphatidylserine (PS)-deficient sEV subpopulations as a major somatic cell-derived sEV subpopulation in blood because of long blood circulation half-life through escape from macrophage uptake. PS^(−)^-sEVs were identified in various cultured cells as a minor population. However, as a result of rapid uptake of PS^(+)^-sEVs by macrophages, circulating somatic cell-derived sEVs in the blood were found to be mainly PS^(−)^-sEVs. These results suggest that endogenous PS^(−)^-sEVs could indeed be the key player in sEV-mediated intercellular communication, a good target for sEV-based diagnosis, and a potent candidate for sEV-based drug delivery. Our findings bring a paradigm shift in the understanding of the biology and translational applications of sEVs.

## Highlights

• PS^(−)^-sEV was found in various cell line and in plasma
• PS^(−)^-sEV showed extremely long blood retention time
• PS^(−)^-sEVs could indeed be important in sEV-mediated intercellular communication

## Graphical abstract

**Figure:**
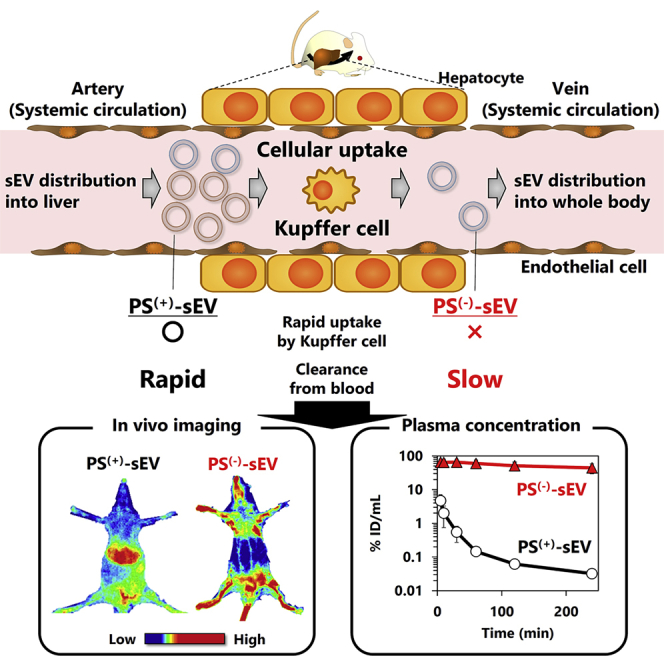

## Introduction

Over the last decade, there has been increasing interest in small extracellular vesicles (sEVs), natural carriers of genetic materials, and proteins (Imai et al., 2015; Morishita et al., 2017; Théry et al., 2018). To understand the molecular mechanism of sEV-mediated intercellular communication, it is essential to shed light on the pathophysiological functions and translational applications of sEVs, including their use and/or analysis. However, it was unknown how sEVs are transferred to remote cells *in vivo* despite the fact that sEVs are rapidly cleared from the blood with an elimination half-life of less than 10 min (Imai et al., 2015; Charoenviriyakul et al., 2017; Morishita et al., 2017). As recently highlighted by many researchers, cells can release heterogeneous sEVs with distinct biological and physicochemical properties (Kowal et al., 2016; Zhang et al., 2018; Matsumoto et al., 2020). If sEVs subpopulation with long half-life exist, they could be the key player. However, there have been no reports about this hitherto.

Previously, we have comprehensively screened sEV surface molecules responsible for rapid blood clearance of sEVs (Matsumoto et al., 2017b; Charoenviriyakul et al., 2018). We identified phosphatidylserine (PS), a negatively charged phospholipid abundantly contained in the sEV membrane (Subra et al., 2007; Llorente et al., 2013), as the key molecule (Matsumoto et al., 2017b, 2020). Thus, assuming that sEVs with no PS on the outer leaflet of the membrane (PS-deficient sEV; PS^(−)^-sEVs) subpopulations exist in the bulk sEV population, we hypothesized that PS^(−)^-sEV could circulate in the blood with a long half-life.

Here, we brought a paradigm shift by determining PS^(−)^-sEVs, a novel sEV subpopulation that could be the key player in sEV-mediated genetic exchange *in vivo*. We identified PS^(−)^-sEVs in various cultured cell-derived sEVs as minor subpopulations, whereas sEVs in endogenous somatic cell-derived sEVs in the blood were found to be mainly PS^(−)^-sEV due to long-circulation time.

## Results

### PS^(−)^-sEV derived from B16BL6 cells showed prolonged blood circulation and little liver accumulation

**Figure 1:**
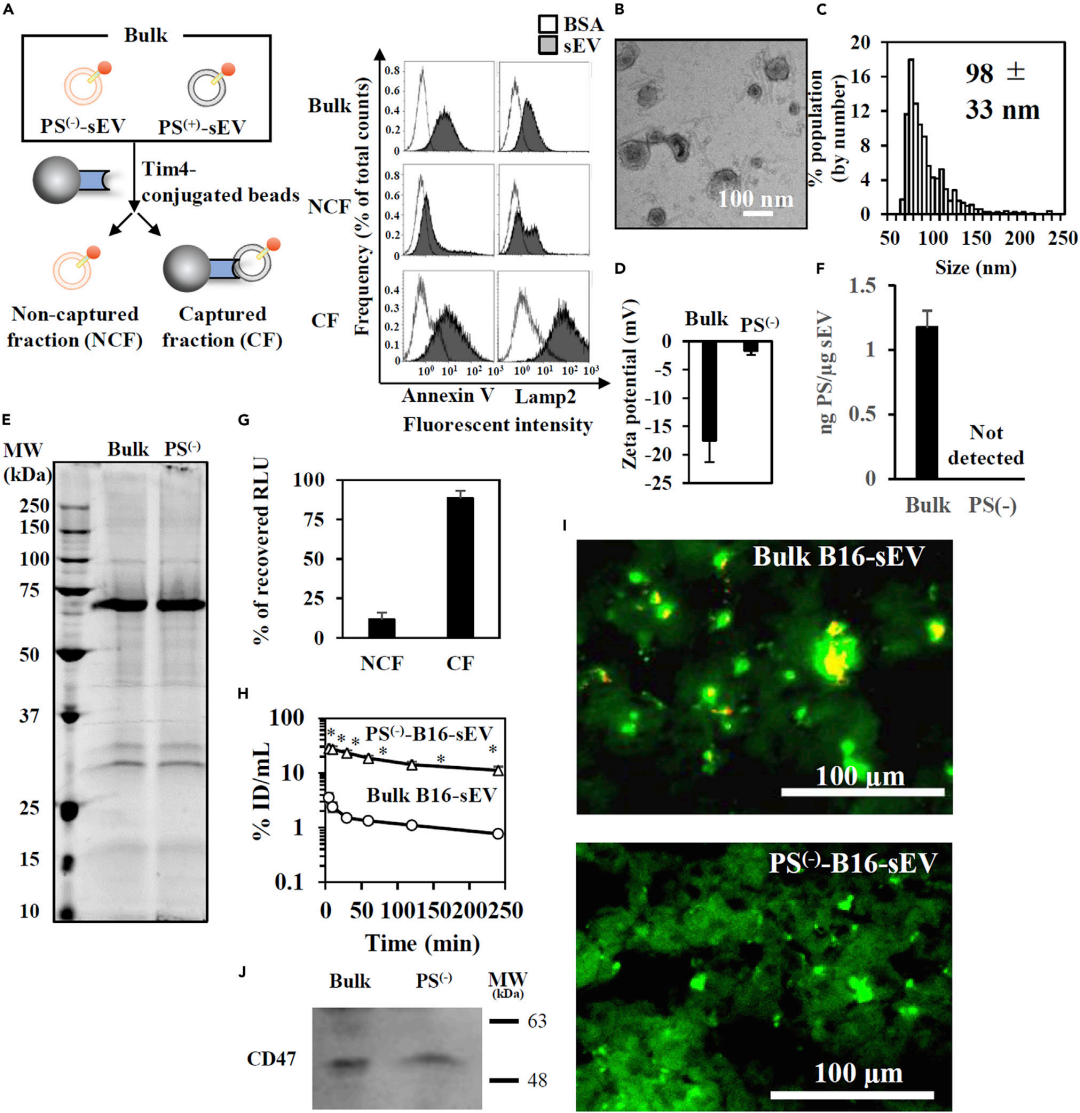
Identification of a distinct PS^(−)^-sEV subpopulation in B16-derived sEVs and its *in vivo* fate after iv. administration (A)-(F) (A) Left: Scheme of the separation of bulk sEVs into PS^(−)^-sEVs (NCF) and PS^(+)^-sEVs (CF) by Tim4-based affinity capturing. Right: Detection of PS or Lamp2 on sEV-gLuc Ab-conjugated bead. BSA was set as a control sample for sEV comparisons. (B–E) (B) morphology (scale bar: 100 nm), (C) size distribution, (D) zeta potential, and (E) protein compositions of gLuc-labeled PS^(−)^-sEVs in NCF Results are expressed as the mean ± SD (n = 3). (F) PS amount of bulk and PS^(−)^-sEVs measured by ELISA. PS amount of PS^(−)^-sEV was below the detection limit (0.62 ng PS/μg sEV). (G) The balance of recovered gLuc enzyme activity of NCF and CF. (H) Time course of serum concentrations of gLuc activity after iv. administration of bulk B16-sEV (1.5×10^9^ RLU/10s/dose) or PS^(−)^ B16-sEV (2.6×10^9^ RLU/10s/dose) labeled with gLuc. ID: injected dose. ∗p < 0.05 versus bulk B16-sEV by Student's t-test. (I) Cellular uptake of bulk or PS^(−)^-sEVs labeled with PKH26 in the liver at 12 hr after iv. administration (approximately 1 μg/dose). Green: F4/80. Red: sEVs. Yellow signal indicates colocalization of sEV and macrophages. Scale bar: 100 μm. (J) CD47 expression in sEVs detected by western blotting.

gLuc-Lamp2c pDNA, a plasmid DNA encoding *Gaussia* luciferase (gLuc: a reporter protein) fused with Lamp2c, a protein abundantly found in sEV membranes (Alvarez-Erviti et al., 2011; Théry et al., 2018), was newly constructed. At first, gLuc-labeled bulk sEVs were collected from B16BL6 (B16) cells transfected with gLuc-Lamp2c pDNA (Figures S1A–S1E). Bulk gLuc-labeled sEVs were rapidly cleared from blood circulation after intravenous (i.v.) administration in macrophage-dependent manner (Figure S1F). To assess whether a PS^(−)^-sEV subpopulation exists, we fractionated bulk B16-derived sEV into PS^(+)^- or PS^(−)^-sEV subpopulations by incubating with Tim4 (high affinity to PS)-conjugated magnetic beads (Figure 1A) (Yoshida et al., 2017). The removal of PS^(+)^−sEVs from the non-captured fraction (NCF), as well as the presence of PS^(−)^-sEVs in the NCF was confirmed by the observation of spherical vesicles with weak negative surface charge (−1.72 ± 0.74 mV) and measurement of an average size of 100 nm, and protein profile similar to bulk sEVs (Figures 1B–1E). In addition, PS amount of PS^(−)^-sEV was at least 2-fold lower than that of bulk sEV although accurate amount of PS in PS^(−)^-sEV could not be measured because of the low amount of PS in PS^(−)^-sEV (Figure 1F). gLuc activity suggests that PS^(−)^-sEV was a minor sEV subpopulation that consists approximately 10% of total sEV (Figure 1F). In addition, PS^(−)^-sEVs were also identified in another 6 different cultured cells (Figure S2). Next, we evaluated the *in vivo* fate after i.v. administration. Blood clearance of PS^(−)^-sEVs was much slower than that of bulk sEVs (Figure 1G, Table S1). While bulk sEVs accumulated in the liver and spleen due to macrophage uptake, PS^(−)^-sEVs were hardly detected in both organs (Figures 1H and S3). Instead, PS^(−)^-sEVs, as well as bulk sEVs, were taken up by vascular endothelial cells in the lung (Figure S3). Expression level of CD47, which is known to work as “Not eat me” signal, was comparable between bulk and PS^(−)^-sEV (Figure 1J), which implies that CD47 content in the sEVs was not so large enough to prevent sEVs from the recognition by macrophages. These results suggest that the weak negative charge of PS^(−)^-sEV is a key factor for prolonged blood circulation and little liver accumulation.

Since PS^(−)^-sEV showed weaker negative charge than bulk sEVs, anion exchange chromatography (Anion-EC) was utilized to investigate whether PS^(−)^-sEVs can be isolated from the bulk sEVs by a method other than affinity-based methods (Figure S4A). As expected, sEVs eluted in the flow-through fraction was PS-negative and possessed weak negative charge (Figures S4B–S4E). Moreover, sEVs eluted in the flow-through fraction showed a comparable blood clearance to that of sEVs isolated through Tim4-beads (Figure S4F).

### PS^(−)^-sEV derived from B16 cells became major B16-derived sEV subpopulation in blood of B16-bearing mice due to slow clearance

**Figure 2:**
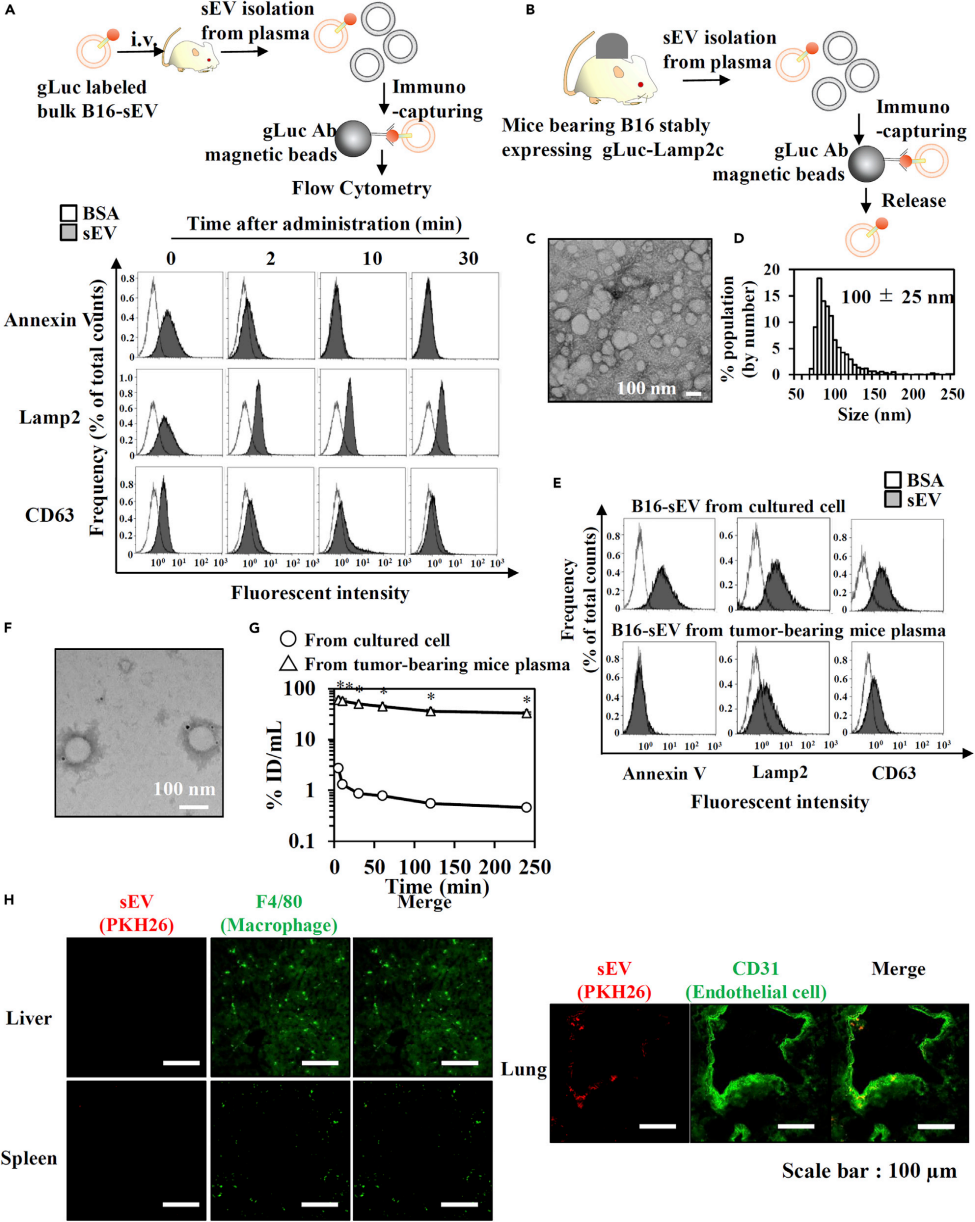
Blood-circulating tumor-derived sEVs were PS^(−)^ and accumulated in the lung after a long time of blood circulation (A) Upper: Experimental scheme. Lower: Flow cytometry analysis of surface markers of gLuc-labeled B16-sEVs in mice plasma at the indicated time points after administration. (B) Scheme of the collection of gLuc-labeled sEV from plasma of mice bearing B16 stably expressing gLuc-Lamp2c. (C and D) The (C) morphology (scale bar: 100 nm) and (D) size distribution of the collected. (E) Detection of surface markers of B16-derived sEVs collected from cultured B16 cells or plasma of B16-bearing mice. (F) TEM observation of B16-sEVs in mice plasma stained with protein A-gold nanoparticles after reacting with an anti-CD146 Ab. Scale bar: 100 nm. (G) Time course of serum concentrations of gLuc activity after iv. administration of B16-derived sEVs collected from cultured B16 cells (3.8 × 10^9^ RLU/10s/dose) or plasma of B16-bearing mice (3.6 × 10^7^ RLU/10s/dose) into mice. Results are expressed as the mean% ID/mL ± SD (n = 3). ∗p < 0.05 versus sEVs from cultured B16 cells by Student's t-test. (H) Cellular uptake of B16-derived sEVs collected from plasma of B16-bearing mice 12 hr after iv. administration into mice. Scale bar: 100 μm.

From the fact that B16-derived PS^(−)^-sEVs showed slow blood clearance, it was hypothesized that PS^(−)^-sEVs become major sEV subpopulation in blood of B16-bearing mice. To evaluate this hypothesis, the population of sEV in the blood of mice receiving i.v. injection of bulk sEV collected from cultured B16 cells was evaluated sequentially after the injection. Most of the PS^(+)^-sEVs in the bulk fraction appeared to be removed from the plasma within as early as 10 min after the injection (Figure 2G). We then evaluated circulating tumor cell-derived sEVs from tumor-bearing mice prepared by inoculation of B16 cells stably expressing gLuc-Lamp2c (Figures 2B–2F). Circulating tumor cell-derived sEVs immunocaptured by gLuc antibody (Ab)-coated beads was 100 nm in diameter and possessed less PS exposure compared with that of bulk sEVs collected from cultured cells. Moreover, blood clearance of circulating tumor cell-derived sEVs after i.v. injection was much slower than that of bulk sEVs collected from cultured cells (Figure 2G). Circulating tumor cell-derived sEVs were taken up by vascular endothelial cells in the lung but scarcely accumulated in the liver and spleen (Figure 2H). These results suggest that PS^(−)^-sEV derived from B16 cells became major B16-derived sEV subpopulation in blood of B16-bearing mice due to slow blood clearance.

### Blood-circulating hepatocyte-derived sEVs stably labeled with gLuc-Lamp2c were negative for PS

**Figure 3:**
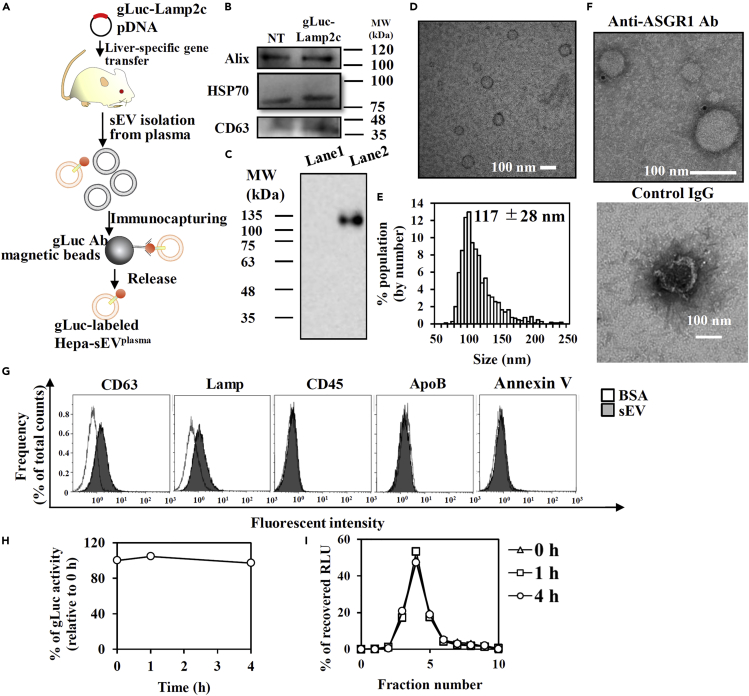
Preparation and characterization of gLuc-Lamp2c-labeled Hepa-sEV^plasma^ (A) Scheme of the preparation of gLuc-labeled Hepa-sEVs^plasma^. (B) Western blotting analysis of sEV marker proteins (CD63, Alix, and HSP70) in sEVs derived from NT and gLuc-Lamp2c transgene mice. (C) gLuc zymography of indicated sEVs. Lane 1; Plasma-sEV, and Lane 2; gLuc-labeled Hepa-sEV^plasma^. (D–F) Characteristics of gLuc-labeled Hepa-sEV^plasma^ immunocaptured by gLuc Ab-coated magnetic beads: (D) sEV morphology by TEM analysis (scale bar: 100 nm), and (E) size histogram measured by qNano instrument. (F) The eluate sEV was stained with protein A-gold nanoparticles after reacting with an anti-ASGR1 Ab (above) or control IgG (below). The samples were observed by TEM. Scale bar: 100 nm. (G) gLuc-labeled Hepa-sEV^plasma^ immunocaptured by gLuc Ab-coated magnetic beads were stained with the indicated Abs and analyzed by flow cytometry. (H and I) sEV labeling by gLuc-Lamp2c proteins and stability in mouse serum. (H) Time course of gLuc activity (H) and SEC analysis (I) of gLuc-labeled Hepa-sEV^plasma^ incubated with 10% mouse serum in PBS at 37°C.

Next, we further investigated whether PS^(−)^-sEV subpopulation derived from somatic cells other than tumor cells become major somatic cell-derived sEV subpopulation in the blood. We decided to label and isolate blood-circulating endogenous hepatocyte-derived sEVs from plasma (Hepa-sEVs^plasma^) with gLuc-Lamp2c probe by using hydrodynamic injections, hepatocyte-specific *in vivo* gene transfer method (Figure S6) (Zhang et al., 1999). We isolated gLuc-labeled Hepa-sEVs^plasma^ from the plasma of mice who received gLuc-Lamp2c pDNA (Figure 3A). The successful isolation of gLuc-labeled Hepa-sEVs^plasma^ and stable gLuc labeling in mouse serum was confirmed (Figures 3, S7, and S8). Detection of asialoglycoprotein receptor 1 (ASGR1; specific hepatocyte marker) in gLuc^+^-sEVs suggested successful gLuc labeling of Hepa-sEVs^plasma^ (Figure 3F). The specificity of gLuc labeling of Hepa-sEVs^plasma^ was also supported by immunoprecipitation assay and labeling assay, revealing that gLuc-Lamp2c scarcely labeled other majorly co-isolated contaminants [*e.g.* LDL particles (ApoB^+^) nor hematopoietic cell-derived-sEVs (CD45^+^)] (Figures 3G, S6, and S7). Moreover, Hepa-sEVs^plasma^ was negative for PS as demonstrated by Annexin V assay (Figure 3G), which indicates that PS^(−)^-sEV subpopulation derived from hepatocytes exists as major hepatocyte-derived population in the blood.

### Blood-circulating hepatocyte-derived sEV without PS exposure showed prolonged blood clearance and little liver accumulation after i.v. injection

**Figure 4:**
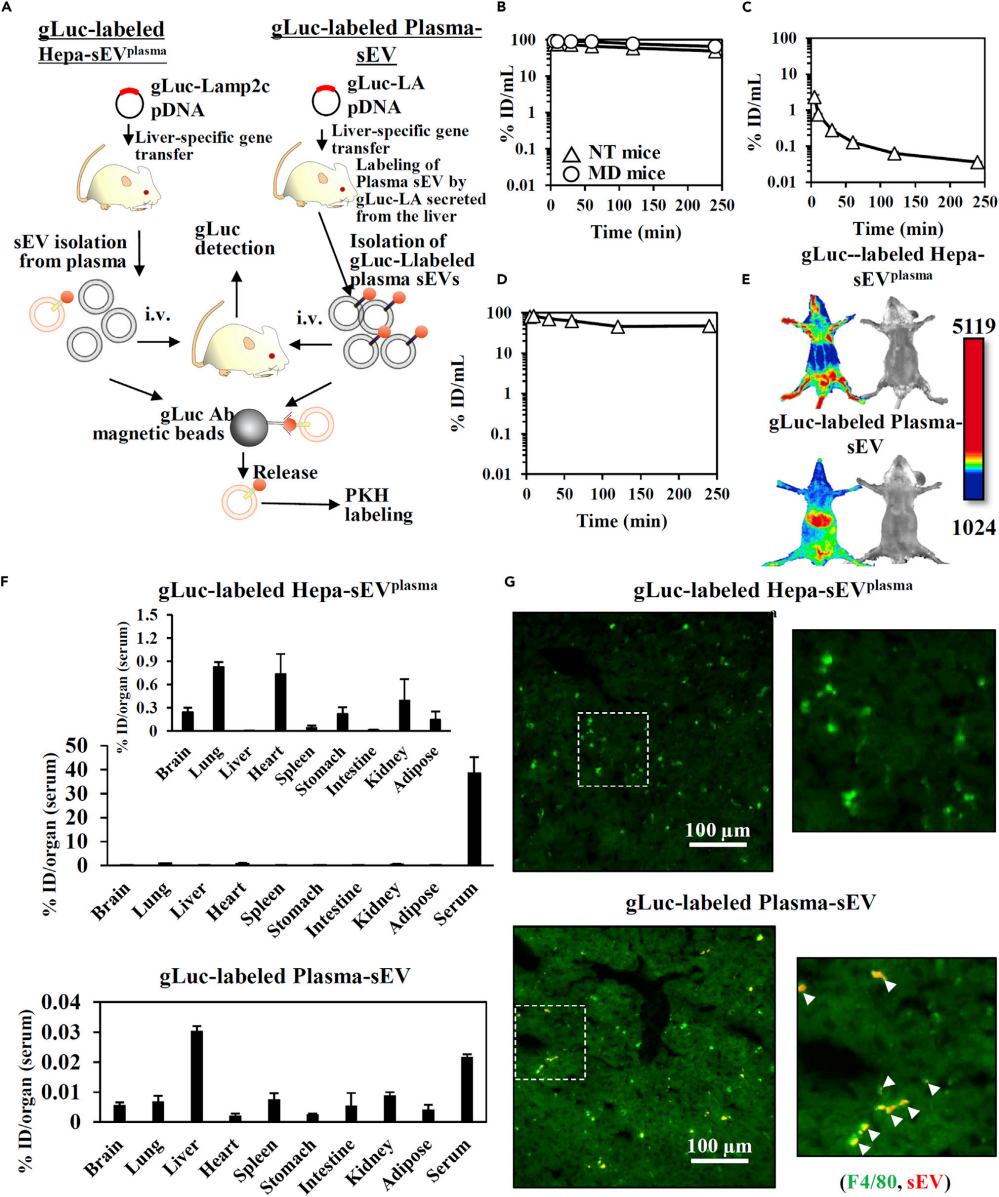
Visualization of the *in vivo* fate of gLuc-Lamp2c-labeled Hepa-sEV^plasma^ and gLuc-LA-labeled Plasma-sEV after iv. administration (A) Experimental procedure to evaluate the *in vivo* fate of gLuc-labeled Hepa-sEV^plasma^ and gLuc-labeled Plasma-sEV. (B–D) Time course of serum concentrations of gLuc enzyme activity after iv. administration of (B) gLuc-labeled Hepa-sEV^plasma^ (2.4×10^8^ RLU/10s/dose), (C) gLuc-labeled Plasma-sEV (2.4×10^10^ RLU/10s/dose) and (D) CD63-gLuc-labeled Hepa-sEV^plasma^ (1.6×10^7^ RLU/10s/dose) into NT mice (triangle) or MD mice (circle). Results are expressed as the mean% ID/mL ± SD (n = 3). (E) Mice were treated with gLuc-labeled Hepa-sEV^plasma^ (1.1×10^10^ RLU/10s/dose) or gLuc-labeled Plasma-sEVs (1.3×10^10^ RLU/10s/dose). The indicated sEVs were imaged 5 min after iv. administration of the sEV samples. (F) Tissue distribution of gLuc activity 12 hr after iv. administration of gLuc-labeled Hepa-sEV^plasma^ (1.6×10^10^ RLU/10s/dose) or gLuc-labeled Plasma-sEV (2.0×10^10^ RLU/10s/dose) into mice. The results are expressed as mean ± SD (n = 3). (G) Cellular uptake of PKH26-labeled sEVs (5 μg/dose) in the liver at 12 hr after iv. injection. The liver section was stained with F4/80-specific Ab and observed by fluorescence microscopy. Right image: the indicated white regions were enlarged. Scar bar: 100 μm.

We evaluated the *in vivo* fate of gLuc-labeled Hepa-sEVs^plasma^ after i.v. injection into mice. gLuc-lactadherin (LA; sEV-tropic protein which enables labeling >90% of the total sEV in the blood (Matsumoto et al., 2020)) was used to prepare gLuc-labeled plasma-sEVs as control (Figure 4A). The blood clearance of gLuc-labeled Hepa-sEVs^plasma^ was much slower compared to that of gLuc-labeled plasma-sEVs (Figures 4B and 4C) as supported by the change of the pharmacokinetic parameters (Table S2). Macrophages were hardly involved in the clearance of gLuc-labeled Hepa-sEVs^plasma^ from the blood (Figure 4B), whereas gLuc-labeled Plasma-sEVs were rapidly cleared from the blood in a macrophage-dependent manner (Figure 4C). This long blood circulation time was independent of the gLuc-Lamp2c probe because the same result was also observed with newly designed CD63-gLuc probe, a chimeric gLuc protein fused with the sEV marker CD63 (Figure 4D, Table S2). Compared wiith plasma-sEVs, Hepa-sEVs^plasma^ scarcely accumulated in the liver, which was due to scarce uptake by macrophages (Figures 4E–4G, S9, and S10). Instead, uptake of Hepa-sEVs^plasma^ was observed by vascular endothelial cells in the lung (Figure S10). Taken together, our results suggest that Hepa-sEVs^plasma^ showed enhanced blood circulation with little liver accumulation through escape from macrophage uptake in the liver.

### Macrophage-dependent *in vivo* selection of PS^(−)^-sEV subpopulation in the blood

**Figure 5:**
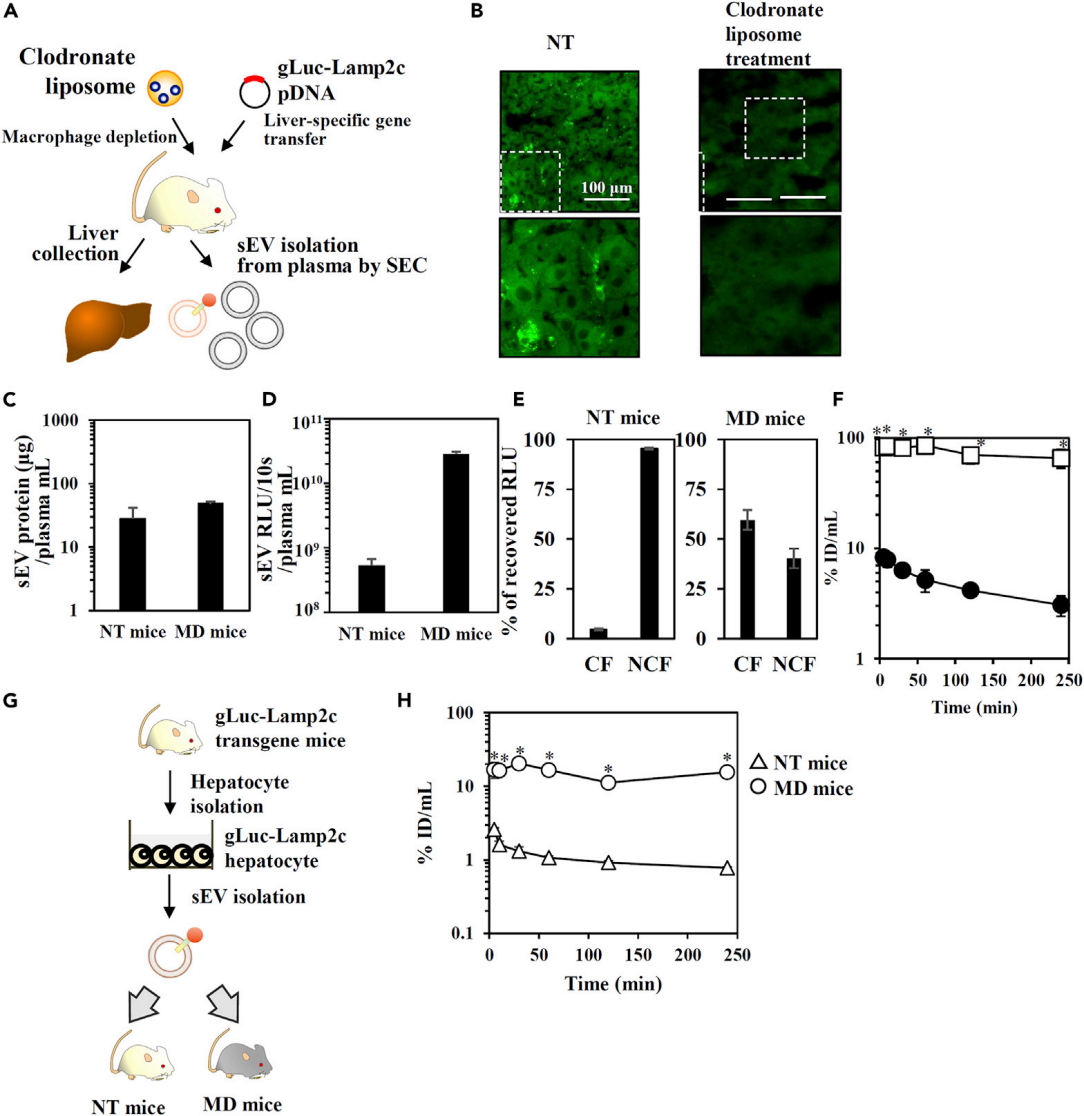
Macrophage-dependent *in vivo* selection of long circulating Hepa-sEV^plasma^ (A) Scheme of the preparation of liver samples and gLuc-labeled Hepa-sEV^plasma^ derived from mice with macrophage depletion. (B) Immunofluorescence staining of liver macrophages after clodronate-encapsulated liposome treatment. Upper images: the green channel corresponds F4/80-specific Ab-derived signals. Lower images: the indicated white regions of the upper images were enlarged. Scale bar: 100 μm. (C and D)(C) The protein amount in sEV-enriched SEC eluate per plasma (mL), and (D) the gLuc enzyme activity of the sEV-enriched SEC eluate per plasma (mL) from mice with partial or complete macrophage depletion was analyzed. Results are expressed as the mean ± SD (n = 3). (E) gLuc-labeled Hepa-sEV^plasma^ collected from NT mice or MD mice were loaded onto Tim4-coated magnetic beads. Next, the balance of gLuc enzyme activity in the NCF and CF was analyzed. Results are expressed as the mean ± SD (n = 3). (F) Time course of serum concentrations of gLuc activity after iv. administration of the indicated gLuc-labeled Hepa-sEV^plasma^ collected from NT mice (open square) or MD mice (closed circle) into mice. Results are expressed as the mean% ID/mL ± SD (n = 3). ∗p < 0.05 versus MD mice Hepa-sEV^plasma^ group by Student's t-test. (G) Scheme of the preparation of gLuc-labeled Hepa-sEV from cultured hepatocytes. (H) Time course of serum concentrations of gLuc activity after the iv. administration of gLuc-labeled Hepa-sEV collected from cultured hepatocytes (3.4×10^8^ RLU/10s/dose) into NT mice or MD mice. Results are expressed as the mean% ID/mL ± SD (n = 3). ∗p < 0.05 versus NT mice by Student's t-test.

Considering the heterogeneous nature of sEVs, we next verified the hypothesis that Hepa-sEVs^plasma^ is a long circulating subpopulation which was *in vivo* selected by macrophages. We prepared gLuc-labeled Hepa-sEVs^plasma^ derived from gLuc-Lamp2c-transferred mice with macrophage depletion using clodronate liposome treatment (Figures 5A and 5B). Macrophage depletion increased the ratio of PS-positive sEVs in Hepa-sEVs^plasma^ as demonstrated by Tim-4-beads assay and led to the 30-fold increased level of Hepa-sEVs^plasma^, which was a large difference compared to that of total sEV-related protein (Figures 5C–5E). The Hepa-sEVs^plasma^ from macrophage depletion mice were rapidly cleared from the blood circulation after i.v. administration compared with Hepa-sEVs^plasma^ from NT mice, which was a clear difference compared to results obtained by using gLuc-labeled plasma-sEVs collected from macrophage-depleted or NT mice (Figure 5F, Table S3). These results indicated that macrophage depletion led to abundant contamination of the PS-exposed Hepa-sEV^plasma^ subpopulation with short blood circulation. In another set of experiments, the total Hepa-sEV population labeled with gLuc was isolated from the primary hepatocytes from mice receiving gLuc-Lamp2C gene transfer (Figures 5G and S11). Macrophage-dependent rapid clearance of Hepa-sEVs collected from cultured hepatocytes was observed (Figure 5H). These results indicated that the Hepa-sEV^plasma^ subpopulation with a long blood circulation is *in vivo* selected by macrophages from the total Hepa-sEV population.

To verify the involvement of other factors than PS in the selection of the Hepa-sEV^plasma^ by macrophages, the protein compositions of gLuc-labeled Hepa-sEVs^plasma^ from non-treated mice (NT_Hepa), gLuc-labeled plasma-sEVs from NT mice (NT_Plasma), and gLuc-labeled Hepa-sEVs^plasma^ from macrophage-depleted mice (MD_Hepa), which showed different pharmacokinetics, were analyzed by unbiased proteomic profiling (Figure S12) because proteins, CD47 as an example, can be involved in the recognition of sEVs by macrophages. However, proteins that had been shown to affect macrophage uptake were not identified in the analysis. On the contrary, CD36, annexin A5, and beta-2-glycoproteins, which all have high affinity for PS, were reduced in NT_Hepa, which were not recognized by macrophages, compared with the other two groups that were recognized by macrophages. These results suggest that PS is a key factor for the selection of the sEV subpopulation in the blood.

## Discussion

The discovery of a sEV subpopulation that can explain the underlying sEV-mediated genetic exchange mechanism *in vivo* is attracting huge interest (Hoshino et al., 2015; Morishita et al., 2017; Murphy et al., 2019) and stimulates further advances in sEV research. In the present study, we identified a novel PS^(−)^-sEV subpopulation, secreted from various cells, with a long blood circulation time. We also identified a distinct biodistribution accompanied by little uptake of PS^(−)^-sEV by macrophages in the liver.

PS has been recognized as one of the most typical sEV markers (Théry et al., 2018; Van Niel, D'Angelo and Raposo, 2018). Thus, we consider that the identification of PS^(−)^-sEVs is unique and important, and as such can impact the sEV research field. Some recent studies reported the potential existence of PS^(−)^-sEVs in a mixture of PS^(+)^-sEVs (Arraud et al., 2014; Matsumura et al., 2019). However, our findings greatly advance the understanding of PS^(−)^-sEVs because we characterized PS^(−)^-sEVs, which is a minor population *in vitro*, as major somatic cell-derived sEV subpopulation in the blood by analyzing their biodistribution and pharmacokinetics. As for the reason that PS^(−)^-sEVs, which is a minor subpopulation *in vitro*, was a main subpopulation in the blood *in vivo*, slow clearance of PS^(−)^-sEVs can be the reason. For example, in case of B16-derived sEVs, approximately 40-fold slower clearance of PS^(−)^-sEVs compared to bulk sEVs can explain the phenomenon that PS^(−)^-sEVs that consist approximately 10% of bulk sEVs *in vitro* become major B16-derived sEV subpopulation in blood. On the other hand, PS^(+)^-sEV, which is major population in the bulk blood sEV, is probably derived from the cells in the blood such as platelets and erythrocytes (Karimi et al., 2018; Wei et al., 2018).

As PS^(−)^-sEVs showed very unique PK properties, PS^(−)^-sEVs might have very important pathophysiological implications which are worth to re-consider. For example, melanoma-derived sEVs are known to enter the circulation, reach the lung, and build the pre-metastatic niche, resulting in increased metastatic invasion (Peinado et al., 2012; Hoshino et al., 2015). Moreover, tumor-derived sEVs in the blood circulation induce immune suppression through T cell interaction (Hong et al., 2016, 2017). However, these biological roles seem not to be in agreement with the fact that bulk sEVs from melanoma cell lines are rapidly cleared from the blood mainly distributed to the liver (Takahashi et al., 2013; Imai et al., 2015; Charoenviriyakul et al., 2017). Our results, however, could help explain these contradictory observations. Since PS^(−)^-B16-sEVs showed a long blood circulation time and accumulated in the lung (Figure 5), PS^(−)^-B16-sEVs could interact with the circulating T cells and build the pre-metastatic niche of lung. We have also found PS^(−)^-sEVs from a variety of cells. Thus, our study emphasizes the potential impact of novel findings regarding PS^(−)^-sEVs functions on basic sEV research.

sEV-based translational applications include therapeutics or biomarker tests. For therapeutics, attempts have been made to identify artificial regulators to extend the blood circulation time of sEVs (Kooijmans et al., 2016; Kamerkar et al., 2017; Williams et al., 2019). However, the overall extension of t_1/2α_ was extremely limited. PS^(−)^-sEVs can make a paradigm shift as an attractive drug carrier, in terms of their long blood circulation. For example, anticancer agent-loaded PS^(−)^-sEVs modified with tumor targeting ligands could be an efficient and promising therapeutic strategy based on active and passive tumor targeting via enhanced permeability and retention effects (Tian et al., 2014; Kim et al., 2018). Furthermore, sEV-based liquid biopsy could be a promising future approach, especially for early tumor diagnosis (Sun and Liu, 2014; Théry et al., 2018). Purification of somatic cell-derived sEVs from the blood is pivotal for highly sensitive and specific analysis. As somatic cell-derived sEVs in the endogenous blood are mainly PS^(−)^-sEVs, detailed analysis of PS^(−)^-sEVs could allow highly sensitive and specific detection.

In conclusion, we have identified a novel PS^(−)^-sEV subpopulation and characterized their *in vivo* fate, as well as its physicochemical and biological properties. These findings are useful in promoting basic and translational sEV research.

### Limitations of the study

In this study, it was demonstrated that PS^(−)^-sEVs become major somatic cell-derived sEV population in blood due to long blood circulation time of PS^(−)^-sEVs by using three types of mice models, mice with liver-specific gene expression prepared by hydrodynamic injection, mice bearing tumor cells with stable reporter protein expression, and macrophage-depleted mice prepared by clodronate liposome administration. As usage of transgenic mouse systems for labeling somatic cell-derived sEV and preparing macrophage-deficient mice will definitely strengthen the finding, it would be desirable to perform experiments using these mouse model in the future study.

## STAR★Methods

### Key resources table

**Table:** 

REAGENT or RESOURCE	SOURCE	IDENTIFIER
**Antibodies**		

Rabbit anti-gLuc antibody	New England Biolabs Inc	E8023S
Rabbit polyclonal anti-ASGR1 Ab	Proteintech	11739-1-AP
Rabbit anti-CD63 antibody	Santa Cruz Biotechnology	sc-1563
Mouse anti-Alix antibody	BD Biosciences	611620
Rabbit anti-HSP70 antibody	Cell Signaling Technology	4872S
Rabbit anti-ApoB antibody	Novus Biologicals	NB200527
Rabbit anti-Pmel17 antibody	Santa Cruz Biotechnology	sc-377325
Rabbit anti-CD47 antibody	ABclonal	A11382
Rabbit anti-mouse IgG-HRP	Thermo Fisher Scientific	61-0120
Mouse anti-rabbit IgG-HRP	Santa Cruz Biotechnology	sc-2357
Alexa fluor 488-labeled anti-Lamp2 antibody	Thermo Fisher Scientific	53-1072-80
PE-labeled anti-CD63 antibody	Biolegends	143903
PE-labeled anti-CD45 antibody	Biolegends	103105
Alexa fluor 488-labeled anti-rabbit IgG	Abcam	ab150077
Alexa fluor 488-labeled anti-mouse F4/80 antibody	Biolegends	123119
Rabbit anti-mouse CD31 antibody	Biolegends	102501

**Chemicals, peptides, and recombinant proteins**		

Sepharose 2B	Sigma Aldrich	CAS No: 9050-94-6
Protein A/G magnetic beads	Thermo Scientific Fisher	88802
PS Capture™ Exosome Flow Cytometry Kit	Wako Fujifilm	297-79701
PKH26 dye	Sigma Aldrich	PKH26GL-1KT
Macro-Prep®HighQ Media	Bio-Rad	1580040

**Critical commercial assays**		

Mouse Phosphatidylserine (PS) ELISA Kit	MyBioSource, Inc.	MBS2022199
PicaGene Dual Sea Pansy Luminescence Kit	Toyo Ink	PD-11
Bradford assay	Bio-Rad	5000201JA

**Deposited data**		

Proteomic data	This paper	http://proteomecentral.proteomexchange.org PXD019244 (Publicly available)

**Experimental models: Cell lines**		

Mouse: B16BL6 cell	RIKEN BRC	RCB2638

**Experimental models: Organisms/strains**		

C57BL6/J	Japan SLC, Inc.	N/A
Balb/c mice	Japan SLC, Inc.	N/A

**Recombinant DNA**		

Plasmid: gLuc	This paper	N/A
Plasmid: gLuc-LA	This paper	N/A
Plasmid: CD63-gLuc	This paper	N/A
Plasmid: gLuc-Lamp2c	This paper	N/A

**Software and algorithms**		

Perseus software version 1.6.2.2	Max-Planck-Institute of Biochemistry	https://maxquant.net/perseus/
Izon Control Suite software version 3.3	Izon Science Ltd.	https://www.izon.com/
Kaluza software version 1.0	Beckman Coulter	https://www.beckman.com/

### Resource availability

#### Lead contact

Further information and requests for resources and reagents should be directed to and will be fulfilled by the lead contact, Yuki Takahashi (ytakahashi@pharm.kyoto-u.ac.jp).

#### Materials availability

This work did not generate new unique reagents.

#### Data and code availability

Proteomics data have been deposited to the ProteomeXchange Consortium (http://proteomecentral.proteomexchange.org) via the jPOST partner repository (http://jpostdb.org). Accession numbers are listed in the key resources table. Microscopy data and original western blot images reported in this paper will be shared by the lead contact upon request.

Original code was not used in this manuscript.

Any additional information required to reanalyze the data reported in this paper is available from the lead contact upon request.

### Method details

#### Animal

Five-week-old male C57BL6/J or Balb/c mice were purchased from Japan SLC, Inc. (Shizuoka, Japan). The protocols of all the animal experiments were approved by the Animal Experimentation Committee of the Graduate School of Pharmaceutical Sciences of Kyoto University.

#### Plasmid DNA (pDNA)

The coding sequences of gLuc and gLuc-LA were obtained as previously described (Takahashi et al., 2013). The coding sequences of Lamp2c and CD63 were synthesized by FASMAC (Atsugi, Japan). For vector construction, the promoter and enhancer coding sequences of the pCpG-mcs vector (Thermo Fisher Scientific, Waltham, MA, USA) were amplified by PCR and subcloned into the SdaI and HindⅢ sites of pBROAD2-mcs (InvivoGen, San Diego, CA, USA). The chimeric sequences of gLuc-LA, gLuc-Lamp2c, and CD63-gLuc were subcloned into the AflⅡ and Kpnl sites of the constructed vectors encoding the corresponding fusion proteins.

#### sEV isolation from culture medium or mouse plasma

For sEV isolation from B16BL6 murine melanoma cells, cells were obtained and cultured as described previously (Takahashi et al., 2013; Matsumoto et al, 2017a, 2017b). B16BL6 cells were transfected with pDNA using polyethylenimine (PEI) “Max” (Polysciences, Warrington, PA, USA) in accordance with a previous report (Takahashi et al., 2013; Matsumoto et al., 2017a). After transfection, the medium was replaced with Opti-MEM (Thermo Fisher Scientific) and the cells were cultured for 24 h. The conditioned medium was collected and subjected to sequential centrifugation (300 × *g* for 10 min, 2,000 × *g* for 20 min, and 10,000 × *g* for 30 min) to remove cell debris and large vesicles. In addition, the medium was filtered through a 0.2-μm filter. The clarified medium was spun at 100,000 × *g* for 1 h (Himac CP80WX ultracentrifuge, Hitachi Koki, Tokyo, Japan; P50AT2 angle rotor, Hitachi Koki). The supernatant was then collected for subsequent experiments. The pellet was resuspended in phosphate buffered saline (PBS) and spun again at 100,000 × *g* for 1 h. The sEVs were recovered in PBS. For isolation of mouse plasma-derived sEVs (MP-sEV), Balb/c mice were administered the indicated pDNA with a hydrodynamics-based procedure in which pDNA dissolved in 10% volume/body (v/w) weight of saline is injected into the tail vein of mice within 5 sec. Four days after the pDNA administration, the mice were sacrificed and whole blood was collected from the vena cava. The blood was immediately treated with 10% EDTA at a volume ratio of 100 : 1, followed by centrifugation at 8,000 × *g* for 20 min to collect the plasma. The plasma was stored at -80°C until use. The MP-sEVs were isolated based on size exclusion chromatography (SEC), according to a previous report (Matsumoto et al., 2020). In brief, sepharose 2B (Sigma Aldrich, St. Louis, MO, USA) was packed into 1.5 cm × 12 cm mini-columns (Bio-Rad, Herculues, CA, USA; Econo-Pac columns) to make a 10 mL column bed. The column was blocked with 2% bovine serum albumin (BSA) solution and washed with PBS. Next, the filtered plasma sample (1 mL) was loaded onto the column and the eluate was collected (fraction 0). Subsequently, 1 mL of PBS was repeatedly loaded onto the column to collect the following fractions, which were sequentially numbered. Based on the elution pattern of gLuc-Lamp2c-labeled B16BL6-derived sEVs (^gLuc-Lamp2c^B16-sEVs), fraction 4 or 5 was selected to be used as an MP-sEV-enriched fraction.

#### Concentration of sEVs from the SEC eluate

Protein A/G magnetic beads (50 μL; Thermo Scientific Fisher) were incubated with gLuc antibody (Ab) (New England Biolabs Inc., Madison, WI, USA) at a 1:25 dilution for 1 h at room temperature with gentle agitation. After the beads were washed with PBS, they were resuspended in 150 μL of PBS with 80 μg of sEV sample for 1 h incubation. The sEVs captured on beads were magnetically separated and washed with PBS. The sEV-bead complexes were treated with 100 mM glycine buffer (pH 2.0) for 10 min with gentle agitation. Next, the tubes were placed on a magnet and the supernatants were carefully collected. Immediately after the supernatant collection, 250 mM NaOH was added for neutralization.

#### Measurement of PS amount of sEV

The amount of PS in sEVs was measured by Mouse Phosphatidylserine (PS) ELISA Kit (MyBioSource, Inc. San Diego, CA, USA) according to the manufacturer’s protocol with some modification that PBS supplemented with 5% BSA and 0.05%Tween 20 was used as dilution buffer.

#### Characterization of the physicochemical properties

Morphologies, size distribution and surface charges of the sEV samples were evaluated as described previously (Takahashi et al., 2013; Imai et al., 2015; Matsumoto et al., 2017b, 2020). Their morphology was observed by transmission electron microscopy (TEM). The sEV sample was added to an equal volume of 4% paraformaldehyde (Nacalai Tesque, Kyoto, Japan), and the mixture was applied to a carbon formvar film-coated transmission electron microscope grid (ALLIANCE Biosystems, Osaka, Japan). The sample was then washed with PBS and fixed by incubation with 1% glutaraldehyde for 5 min, then washed with distilled water, and incubated with 1% uranyl acetate for 5 min. The sample was observed under a transmission electron microscope (Hitachi H-7650; Hitachi High Technologies Corporation, Tokyo, Japan). For the measurement of size distribution, the qNano instrume (Izon Science Ltd., Christchurch, New Zealand) was used. The NP150 nanopore was used according to the manufacturer’s instructions. All sEV samples and calibration particles (Izon Science Ltd.) were measured at 47.0 mm stretch with a voltage of 0.5-0.8 V. Collected data were processed by Izon Control Suite software version 3.3. For the measurement of surface charge of sEV samples, a Zetasizer Nano ZS (Malvern Instruments, Malvern, UK) was used according to the manufacturer’s protocol.

#### Characterization of protein composition

For detection of the indicated proteins by immunoelectron microscopy, the sEV samples were first fixed with 4% paraformaldehyde in PBS. The sample was then applied to a carbon formvar film-coated transmission electron microscope grid (Alliance Biosystems) and incubated for 20 min. The grid was washed with 50 mM glycine in PBS, blocked with 5% BSA in PBS and incubated with a rabbit anti-gLuc antibody (Ab) (1: 500 dilution; New England Biolabs Inc.) and a rabbit anti-ASGR1 Ab (1: 50 dilution; Proteintech., Rosemont, IL, USA) for 1 h. After washing with 0.5% BSA in PBS, the sample was incubated with a 10 nm protein A-gold conjugate (BB Solution, Cardiff, UK) for 1 h, followed by immerse fixation with 1% glutaraldehyde in PBS. Following washing with distilled water, the grid was stained with uranyl acetate and observed by TEM. Sodium dodecyl sulfate-polyacrylamide gel electrophoresis (SDS-PAGE) of the sEVs samples was performed as described previously *(4)*. For protein staining, the gel was stained with Lumitein^TM^ Protein Gel reagent (Biotium, Inc., Landing Parkway Fremon, CA, USA) according to the manufacturer’s protocol. The stained gel was observed using the LAS-3000 instrument (FUJIFILM, Tokyo, Japan). Western blotting analysis of sEV markers (CD63, Alix, HSP70) and other proteins (calnexin, gLuc, ApoB, gp100, ASGR1, CD47) was conducted as described previously (Matsumoto et al., 2017a). The following primary Abs were used: rabbit anti-CD63 Ab (1:200 dilution; Santa Cruz Biotechnology, Dallas, TX, USA), mouse anti-Alix Ab (1:20000 dilution; BD Biosciences, San Jose, CA, USA), rabbit anti-HSP70 Ab (1:1000 dilution; Cell Signaling Technology, Danvers, MA, USA), rabbit anti-Calnexin Ab (1:1000 dilution; Santa Cruz Biotechnology), rabbit anti-gLuc Ab (1:1000 dilution; New England Biolabs Inc.), rabbit anti-ApoB Ab (1:200 dilution; Novus Biologicals, Littleton, CO, USA), rabbit anti-Pmel17 Ab (1:200 dilution; Santa Cruz Biotechnology), rabbit anti-ASGR1 Ab (1:1000 dilution; Proteintech), and rabbit anti-CD47 Ab (1:500 dilution; ABclonal, MA, USA). The following secondary antibodies were used: rabbit anti-mouse IgG-HRP (1:2000 dilution; Thermo Fisher Scientific), mouse anti-rabbit IgG-HRP (1:1000 dilution; Santa Cruz Biotechnology), and TidyBlot™ Western blot detection reagent (1:100 dilution; Bio-Rad).

#### Flow cytometric assay of sEV-bead complexes

Protein A/G magnetic beads (2.5 μL; Thermo Scientific Fisher) were incubated with gLuc Ab (New England Biolabs Inc.) at a 1:25 dilution for 1 h at room temperature with gentle agitation. After the beads were washed with PBS, they were resuspended in 50 μL of PBS with 2 μg of sEV sample and incubated for 1 h. The sEVs captured on beads were magnetically separated, washed with PBS and resuspended in 500 μL of N-2-hyfroxyethylpiperazine-N’-2-ethanesulfonic acid (HEPES) buffer [10 mM HEPES (pH 7.4), 140 mM NaCl, and 2.5 mM CaCl_2_]. For detection of sEV surface molecules, the sEVs captured on beads were incubated with the indicated fluorescent-labeled protein or Ab for 1 h with gentle agitation. The fluorescent-labeled protein or Abs used were the following: FITC-labeled annexin V (1:25 dilution; Biolegends, San Diego, CA, USA.), Alexa fluor 488-labeled anti-Lamp2 Ab (1:25 dilution; Thermo Fisher Scientific), PE-labeled anti-Lamp2 Ab (1:25 dilution; Biolegends) and PE-labeled anti-CD63 Ab (1:25 dilution; Biolegends), PE-labeled anti-CD45 Ab (1:25 dilution; Biolegends), anti-ApoB Ab (1:100 dilution; Novus Biologicals) + Alexa fluor 488-labeled anti-rabbit IgG (1:1000 dilution; Abcam, Cambridge, UK). After the sEVs captured on beads were washed with PBS, fluorescence was detected by Gallios^TM^ flow cytometry (Beckman Coulter, Brea, CA, USA). Data were analyzed using Kaluza software (version 1.0, Beckman Coulter).

#### Labeling stability of gLuc proteins on sEVs in serum

The sEVs labeled with the indicated chimeric gLuc proteins were incubated in 10% mouse serum in PBS solution at 37°C with gentle agitation. Samples were collected at the indicated time points. The stability of gLuc enzyme activity was evaluated by measuring gLuc enzyme activity in the collected samples. Samples were applied to SEC packed with either sepharose-2B or sephacryl-s300 (GE healthcare; Chicago, IL, USA) was collected (fraction 0). Subsequently, PBS was applied to collect the fraction (1 mL each). The fraction was numbered by collection order and gLuc enzyme activity of each fraction was measured.

#### sEV clearance in blood after i.v. administration

Macrophage-depleted mice were prepared by the administration of clodronate-encapsulated liposome (Imai et al., 2015; Matsumoto et al., 2020). The clearance of gLuc-labeled sEVs from the blood after i.v. administration in mice was measured based on a luciferase activity assay as described previously(Takahashi et al., 2013; Imai et al., 2015; Matsumoto et al., 2017b, 2020). The time course data were analyzed on the basis of a two-compartment i.v. model. The sEV serum concentration is described as a function of time by Equation (1), and the parameters A, B, α, and β in the equation were determined using the nonlinear least-squares program MULTI^6^ to fit a curve to the serum concentration-time profile.

(Equation 1) $${\text{C}}_{\left(t\right)}=A\cdot e^{-\alpha t}\;+\;B\cdot e^{-\beta t}\;$$

The blood elimination half-time in the α phase (t_1/2α_) and β phase (t_1/2β_) were calculated by the determined parameters A, B, α, and β as described previously^4,5^. The area under the curve (AUC), and mean residence time (MRT) were calculated for each animal by integration from 5 to 240 min.

#### PS^(+)^-sEVs capture using Tim4-conjugated beads

After Tim4-conjugated beads (90 μL; Wako Fujifilm, Osaka, Japan) were washed with the wash buffer according to the manufacturer’s instructions, the beads were incubated with 2 μg of sEV sample in 50 μL PBS for 1 h with gentle agitation. The tubes were placed on a magnet and supernatants were carefully collected. The sEVs in the supernatants were characterized and used for the downstream assays.

#### PKH26 labeling of sEVs

For the preparation of PKH26-labeled B16BL6-sEVs, the PKH26 dye (Sigma Aldrich) in a diluent C buffer (Sigma Aldrich) was added to the B16BL6-sEVs and incubated for 5 min at room temperature. To remove the unbound dye, the sEVs + fluorescent dye sample was incubated with 5% BSA in PBS and ultracentrifuged at 100,000 × g for 1 h. The pellet collected contained the PKH26-labeled B16-sEVs. For labeling the indicated chimeric gLuc protein-labeled MP-sEVs loaded onto gLuc Ab-conjugated magnetic beads, PKH26 dye in a diluent C buffer was added to the sEV-bead complexes and incubated for 5 min at room temperature. The tube was placed on a magnet and the sEVs on beads were washed with 5% BSA in PBS followed by washing with PBs 3 times to remove the free dye. MP-sEVs labeled with both PKH26 and chimeric gLuc protein were eluted from the beads as described above.

#### Anion exchange chromatography

Macro-Prep®HighQ Media (Bio-Rad) were packed into 1.5 cm × 12 cm mini-columns (Bio-Rad) to make a 1 mL column bed. The column was washed with PBS. Next, gLuc-Lamp2cB16-sEVs (10-80 μg in 10 mL PBS supplemented with 1% BSA) were loaded onto the column and the flow-through fraction (10 mL) was collected. The flow-through fraction was concentrated by 100 K ultrafiltration (Amicon Ultra, Merck Millipore) before use.

#### Biodistribution of gLuc-labeled sEVs

For gLuc imaging of gLuc-labeled sEVs *in vivo*, mice received an i.v. injection of gLuc-LA or gLuc-Lamp2c-labeled sEVs at a dose of 1×10^10^ RLU/10s/shot, followed by immediate injection of 200 μg of coelenterazine (REGIS Technologies, Morton Grove, IL, USA), a substrate for gLuc, into the tail vein of mice. The imaging was acquired by LAS3000 (Fujifilm). The duration of image acquisition was 5 min. Immediately after chemiluminescence detection, the photographic images of mice were taken to identify the position of the mice. The MultiGauge software (Fujifilm) was used to erase the background to emphasize the distribution of chemiluminescence in mice. For the cellular uptake of gLuc-labeled sEVs in the liver, lung and spleen, mice received an i.v. injection of sEVs labeled with PKH26. After 1 or 12 h of injection, mice were sacrificed for liver, lung, and spleen collection. The harvested organs were frozen at -80°C, and the frozen sections were cut with a freezing microtome (Leica CM3050 S; Leica Biosystems, Germany). The sections were air dried and fixed with 4% paraformaldehyde in PBS. After washing with PBS, sections were stained with Alexa fluor 488-labeled anti-mouse F4/80 Ab (1:50 dilution; Biolegends) or rabbit anti-mouse CD31 Ab (1:50 dilution; Biolegends) + Alexa fluor 488-labeled goat anti-rabbit IgG (1:100 dilution; Abcam) for 1 h at 37°C. The specimens were washed 3 times with PBS and observed under a fluorescence microscope (Biozero BZ-8000; Keyence, Osaka, Japan).

#### Preparation of chimeric protein-enriched sample

The recovered supernatant during sEV isolation from B16BL6 cells, described previously herein, was passed through an Amicon Ultra 100K centrifugal filter (Merck Millipore, Billerica, MA, USA) for gLuc, gLuc-LA and CD63-gLuc protein preparation, and the Nanosep 300K centrifugal device was used (Pall corporation, Port Washington, NY, USA) to remove the remaining vesicles or protein aggregates for gLuc-Lamp2c protein collection. The flow-through medium was then concentrated by ultrafiltration (Amicon Ultra 10K for gLuc protein and Amicon Ultra 30 K for gLuc-LA, CD63-gLuc, and gLuc-Lamp2c). The samples were mixed with a sea pansy luciferase assay reagent (Picagene Dual; Toyo Ink, Tokyo, Japan) and chemiluminescence was measured with a luminometer (Lumat LB 9507; EG&G Berthold, Bad Wildbad, Germany).

#### Ultrafiltration assay

The indicated gLuc-Lamp2c protein or gLuc-Lamp2c-labeled sEVs were applied to the Nanosep 300K centrifugal device (Pall corporation) and centrifuged at 3,000 × *g* for 3 min. The recovered gLuc enzyme activity of the flow-through and filtrate was measured.

#### Isolation of hepatocyte-derived sEVs

Mice anesthetized with isoflurane were kept warm at 37°C with a hot plate during the experiment. The liver was perfused first with Ca^2+^, Mg^2+^-free perfusion buffer (10 mM HEPES, 137 mM NaCl, 5 mM KCl, 0.5 mM NaH_2_PO_4_, and 0.4 mM Na_2_HPO_4_, pH 7.2) for 5 min followed by perfusion buffer supplemented with 5 mM CaCl_2_, 0.05% (w/v) collagenase from Clostridium histolyticum (Sigma Aldrich), and 0.005% (w/v) trypsin inhibitor from soybean (Nacalai Tesque) for 5 min. As soon as the perfusion started, the vena cava and aorta were cut, and the perfusion rate was maintained at 5 mL/min. The liver was then excised, and the cells were dispersed by gentle stirring in ice-cold Hank’s-HEPES buffer. The dispersed cells were filtered through a 40 μm cell strainer, followed by centrifugation at 50 × *g* for 1 min. The pellet was washed twice with Hank’s-HEPES buffer by repeating centrifugation at 50 × *g* for 1 min. The recovered cells were seeded at a density of 4 × 10^6^ cells per 10 cm of culture dish and cultured in RPMI medium (contaminating fetal bovine serum-derived EVs were reduced by centrifugation at 100,000 × *g* for 2 h). The conditioned medium was collected and replaced with fresh RPMI medium every 24 h for three times. The sEVs from the conditioned medium were isolated by an SEC-based method as described above.

#### Proteome analysis

Isolated sEV-related proteins were reduced with 10 mM dithiothreitol (Wako fujifilm) for 30 min, alkylated with 50 mM iodoacetamide (Sigma-Aldrich) for 30 min, and digested with Lys-C (Wako fujifilm, 1:50 enzyme-to-protein ratio) for 3 h followed by trypsin digestion (Promega, 1:50 enzyme-to-protein ratio) overnight in 50 mM ammonium bicarbonate (Wako fujifilm). Digestion was stopped by the addition of trifluoroacetic acid to a final concentration of 0.5%. The peptide mixture solution was desalted with reversed-phase StageTips (Rappsilber et al., 2007) and 1 μg of peptides were injected onto a nanoLC/MS/MS system consisting of an Ultimate 3000 RSLCnano nanoLC pump and Q-Exactive tandem mass spectrometer (Thermo Fisher Scientific). The peptides were separated by a self-pulled analytical column (150 mm length × 100 μm i.d.) packed with ReproSil-Pur C18-AQ materials (3 μm, Dr. Maisch GmbH, Ammerbuch-Entringen, Germany), using a 65-min gradient of 5–40% B (solvent A was 0.5% acetic acid and solvent B was 0.5% acetic acid in 80% acetonitrile) at a flow rate of 500 nL/min. The applied ESI voltage was 2.4 kV and the MS scan range was *m/z* 350–1500 at a resolution of 70,000 (at *m/z* 200) in the orbitrap using an AGC target value of 3 × 10^6^. The top 10 precursor ions were selected for subsequent MS/MS scans. Precursors were fragmented in the HCD (higher-energy collision) cell and analyzed in the orbitrap at a resolution of 17,500 (at *m/z* 200) using an AGC target value of 1 × 10^5^. Dynamic exclusion was applied with an exclusion time of 30 s. Peptides were identified with Mascot version 2.6.1 (Matrix Science, London, UK) against the SwissProt Database (version 2017_04) with a precursor ion mass tolerance of 5 ppm and a product ion mass tolerance of 20 ppm. Up to two missed trypsin cleavages were allowed. Cysteine carbamidomethylation was set as a fixed modification and methionine oxidation was allowed as a variable modification. Peptides were considered identified if the Mascot score was greater than the 95% confidence limit based on the identity score of each peptide. The label-free quantification of peptides was based on the peak area on the extracted ion chromatograms. All of the peptide peak areas were summed for quantification of proteins, and missing values were imputed using Perseus software version 1.6.2.2 (Tyanova et al., 2016). Protein quantitative values were log2-transformed and normalized by subtraction of the median value in each sample. Only proteins with at least two unique peptides were used for further analysis.

#### gLuc-Lamp2c stable B16 tumor MP-sEV isolation

Mice were subcutaneously inoculated with 5 × 10^9^ B16BL6 cells stably expressing gLuc-Lamp2c. Tumor size was measured using a slide caliper, and tumor volume was daily calculated using the following formula: tumor volume (mm^3^) = (longer length × shorter length^2^) × 0.5. When the tumor volume exceeded 1500 mm^3^, the xenograft mice were sacrificed to collect the plasma. MP-sEVs were isolated from the plasma as described above.

### Quantification and statistical analysis

#### Statistical analysis

Differences between two groups and multiple groups were evaluated using the Student's t-test and Tukey-Kramer test, respectively, and a p value of < 0.05 was considered statistically significant.

#### Data availability

Proteomic data have been deposited to the ProteomeXchange Consortium (http://proteomecentral.proteomexchange.org) via the jPOST partner repository (http://jpostdb.org) with the data set identifier PXD019244 (Okuda et al., 2017) and is publicly available.

## Supplemental information

Document S1. Figures S1–S12 and Tables S1–S3

Data S1. Summary of identified proteins by proteomic profiling of sEVs, related to Figure 5
