# Supplementary material for: Phosphatidylserine-deficient small extracellular vesicle is a major somatic cell-derived sEV subpopulation in blood
Source: iScience. 2021 Jul 10;24(8):102839. doi: 10.1016/j.isci.2021.102839 (PMC8326202; doi:10.1016/j.isci.2021.102839)
Supplement: Document S1. Figures S1–S12 and Tables S1–S3 [file mmc1.pdf]

iScience, Volume *24*

Supplemental information

Phosphatidylserine-deficient small extracellular vesicle is a major somatic cell-derived sEV subpopulation in blood

Akihiro Matsumoto, Yuki Takahashi, Kosuke Ogata, Shimpei Kitamura, Naoki Nakagawa, Aki Yamamoto, Yasushi Ishihama, and Yoshinobu Takakura

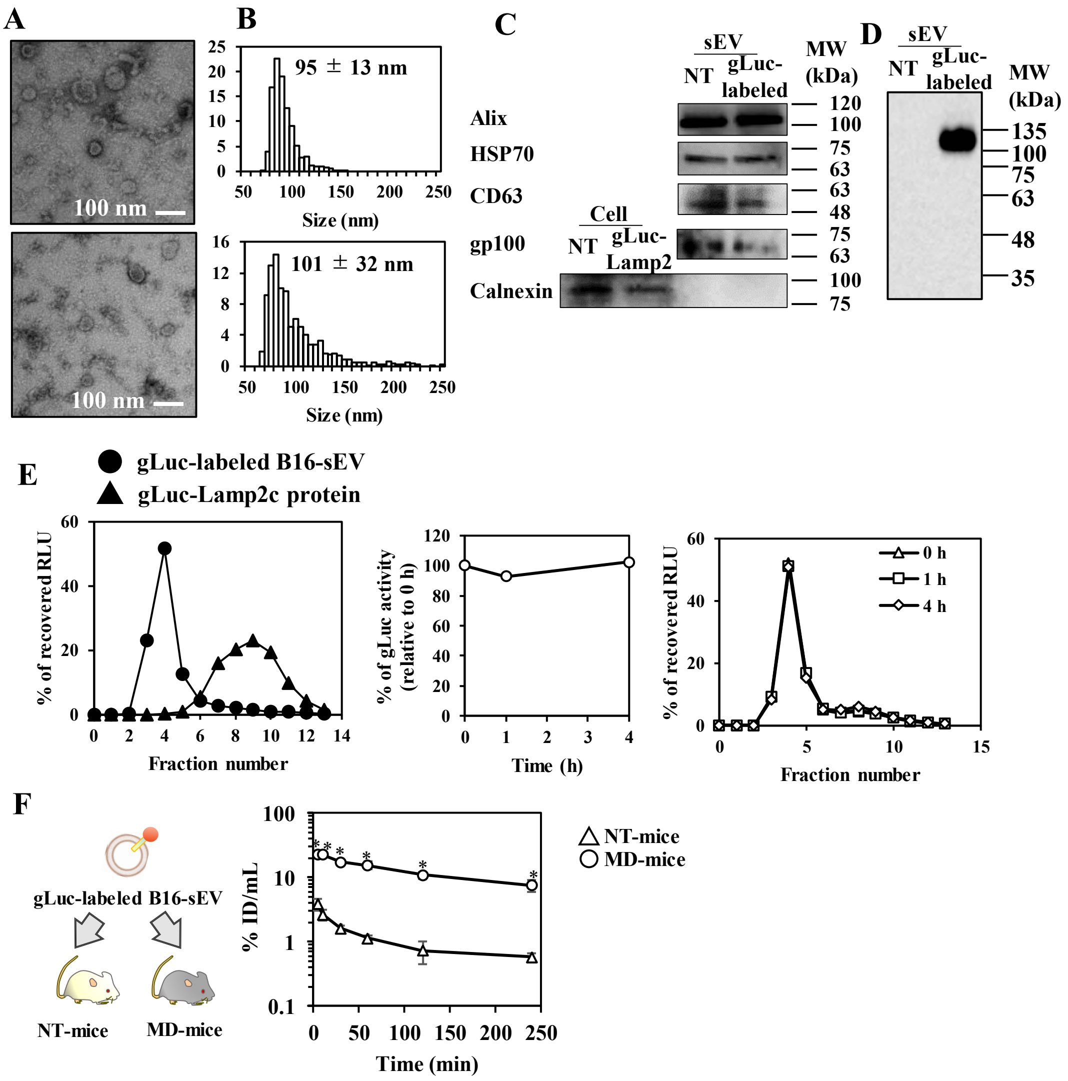

Fig. S1. gLuc-labeled B16-sEVs collected from B16 cells transfected with gLuc-Lamp2c-expressing pDNA were rapidly cleared from the blood in a macrophage-dependent manner, Related to Fig.1. (A) Morphological observation of B16-sEVs (upper) and gLuc-labeled B16-sEVs (lower) by transmission electron microscopy (TEM). Scale bar: 100 nm. (B) Size distribution of B16-sEV (upper) and gLuc-Lamp2c-labeled B16-sEV (lower) measured by qNano instrument. (C) Western blotting analysis of sEV marker proteins (CD63, Alix, and HSP70), a melanoma marker protein (gp100) as well as a negative sEV marker protein (Calnexin). (D) GLuc zymography of indicated B16-sEVs. (E) Labeling stability of gLuc-Lamp2c to B16-sEV in mouse serum. Left; Size exclusion chromatography (SEC) elution pattern of gLuc-Lamp2c protein, and gLuc-labeled B16-sEV. Middle: Time-course of gLuc activity incubated with 10% mouse serum in PBS. Right; SEC analysis of gLuc-labeled B16-sEV incubated with 10% mouse serum in PBS at 37°C for the indicated time periods. (F) Time-course of serum concentrations of gLuc activity after i.v. administration of gLuc-labeled B16-sEV into non-treated (NT)-mice (1.2×10^9 RLUs/10/dose) or macrophage-depleted (MD)-mice (2.2×10^9 RLU/10s/dose). Results are expressed as the mean of the percentage of the administered dose/mL (% ID/mL) ± SD (n = 3). *p < 0.05 versus NT-mice by Student's t-test.

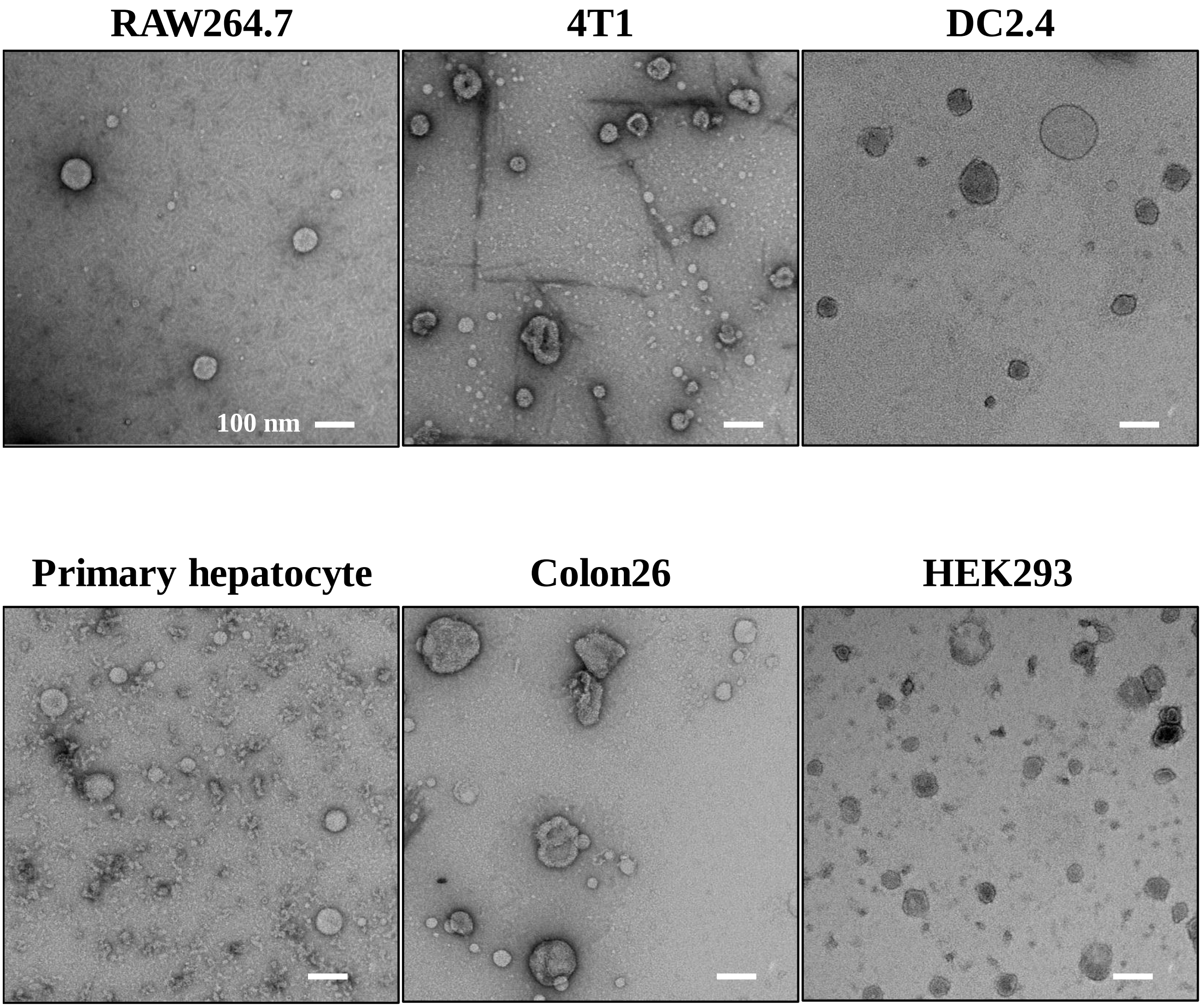

Fig. S2. PS$^{(-)}$-sEVs from 6 different cultured cells were observed by TEM, Related to Fig. 1. RAW264.7 cells: mouse macrophage-like cells. 4T1 cells: mouse breast cancer cells. DC2.4 cells: mouse dendritic cells. Colon26 cells: mouse colorectal cancer cells. HEK293 cells: human embryonic kidney cells. Scale bar: 100 nm.

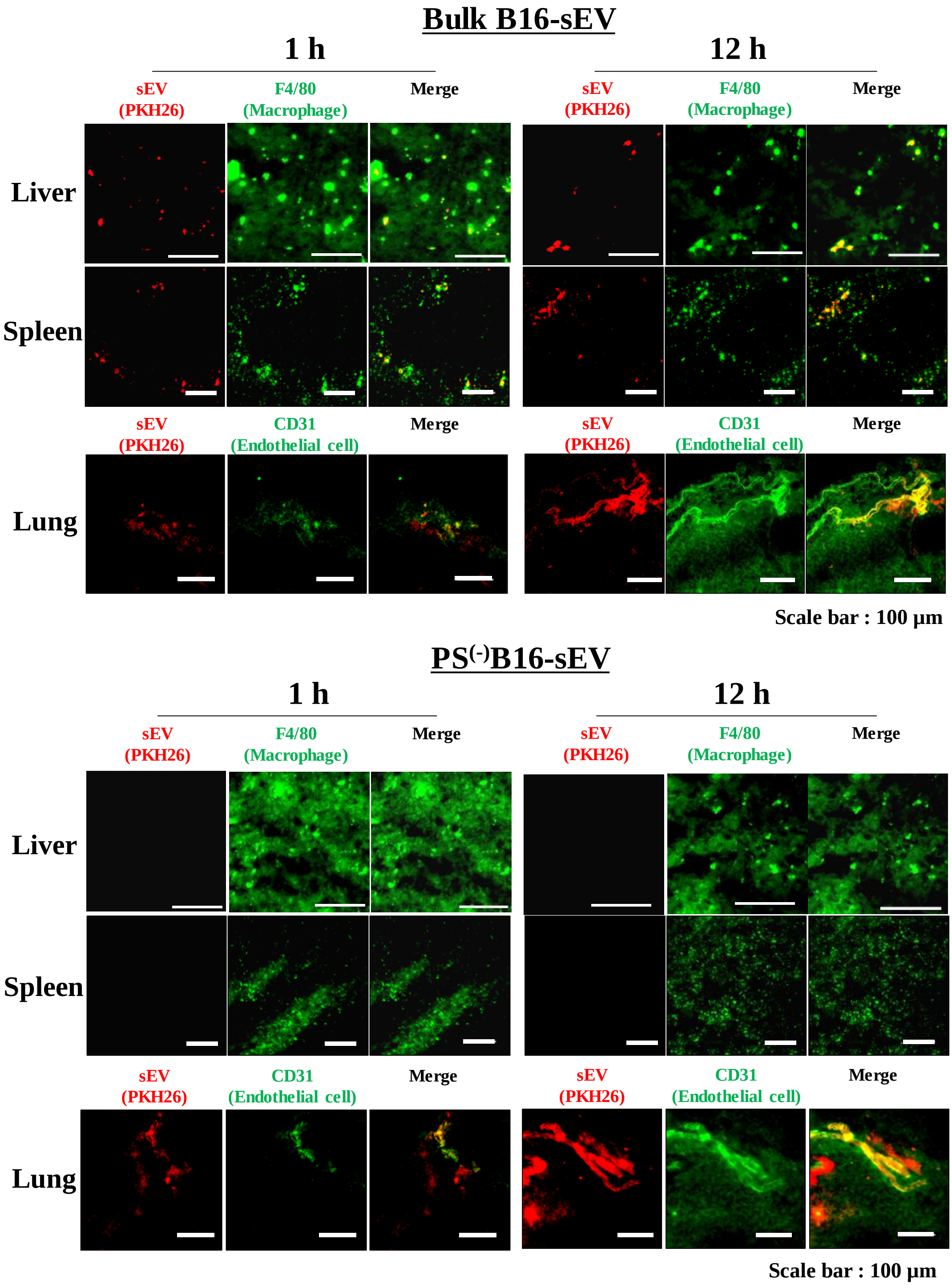

Fig. S3. PS$^{(-)}$-B16-sEVs distributed in the lung, but not in the liver and spleen, Related to Fig.1. The bulk B16-sEVs or PS$^{(-)}$-B16-sEVs were labeled with PKH26, followed by i.v. administration into mice (1 μg/dose). After 4 or 12 h of injection, the liver, spleen, or lung were collected and cut into a cryostat section. The section was stained with F4/80-specific Ab (liver or spleen) or CD31-specific Ab (lung) and observed by fluorescence microscopy. PS, phosphatidylserine, sEVs, small extracellular vesicles; Ab, antibody. Scale bar: 100 μm.

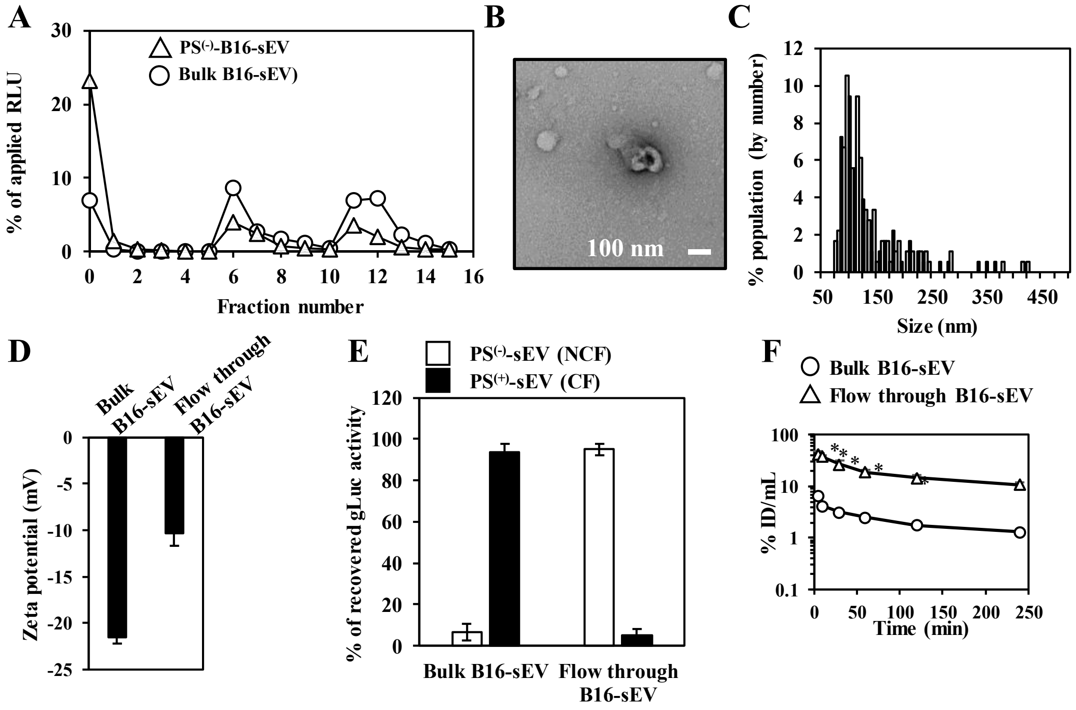

Fig. S4. PS$^{(-)}$-sEV was isolated as flow through fraction after anion exchange chromatography (Anion-EC), , Related to Fig.1. (A) Anion-EC elution profile of bulk and PS$^{(-)}$ B16-sEV labeled with gLuc. The gLuc enzyme activity of each fraction (fraction 0, 10 mL/fraction; fraction 1-15, 1 mL/fraction, respectively) was measured. The flow through fraction (fraction 0) was used as the PS$^{(-)}$-sEV-enriched fraction in the downstream analysis. (B) TEM (scale bar: 100 nm), (C) qNano, and (D) zetasizer analysis was conducted for physicochemical characterization of flow through B16-sEV. Results are expressed as the mean ± SD (n = 3). (E) The balance of PS$^{(-)}$-sEV and PS$^{(+)}$-sEV in the flow through B16-sEV after the separation by Tim4-conjugated magnetic beads. Results are expressed as the mean ± SD (n = 3). (F) Time-course of serum concentrations of gLuc activity after i.v. administration of bulk B16-sEV (9.9×10^8 RLU/10s/dose) or flow through B16-sEV (5.2×10^8 RLU/10s/dose) labeld with gLuc into NT-mice. Results are expressed as the mean % ID/mL ± SD (n = 3). *$p < 0.05$ versus bulk B16-sEV by Student's t-test.

A

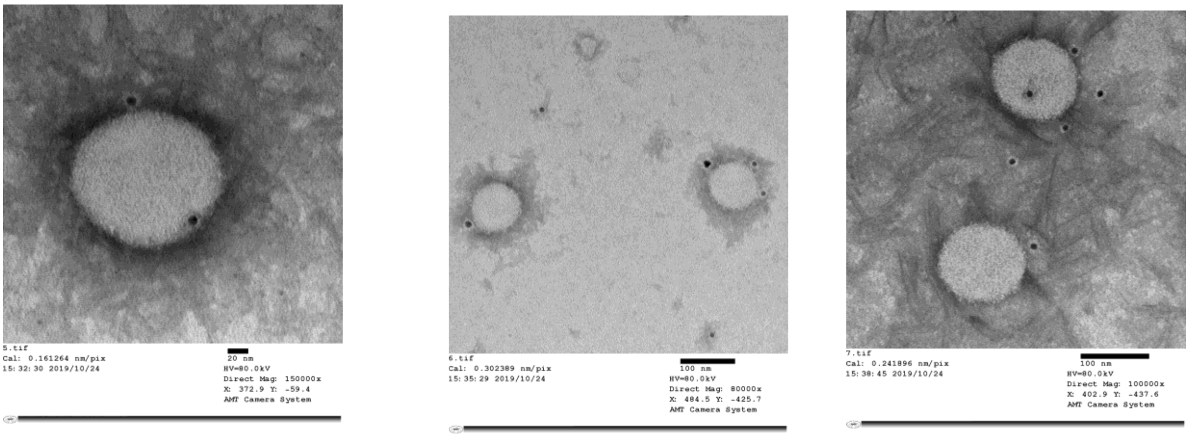

B

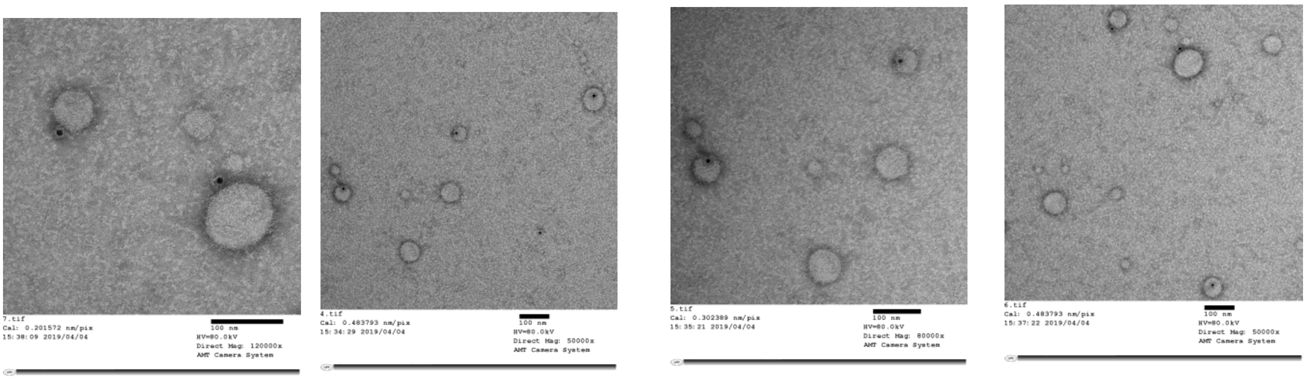

Fig. S5. CD146 and ASGR protein on sEV surface proteins were observed by TEM by staining sEV with protein A-gold nanoparticles after reacting with antibodies, Related to Figs. 2 and 3. (A) TEM observation of B16-sEVs in plasma of tumor-bearing mice stained with protein A-gold nanoparticles after reacting with an anti-CD146 Ab. Additional pictures for Fig.2F. (B) TEM observation of sEVs derived from serum of mice bearing hydrodynamic gene transfer of Lamp2c-gLuc stained with protein A-gold nanoparticles after reacting with an anti-ASGR Ab. Additional pictures for Fig.3F. Scale bar: 100 nm.

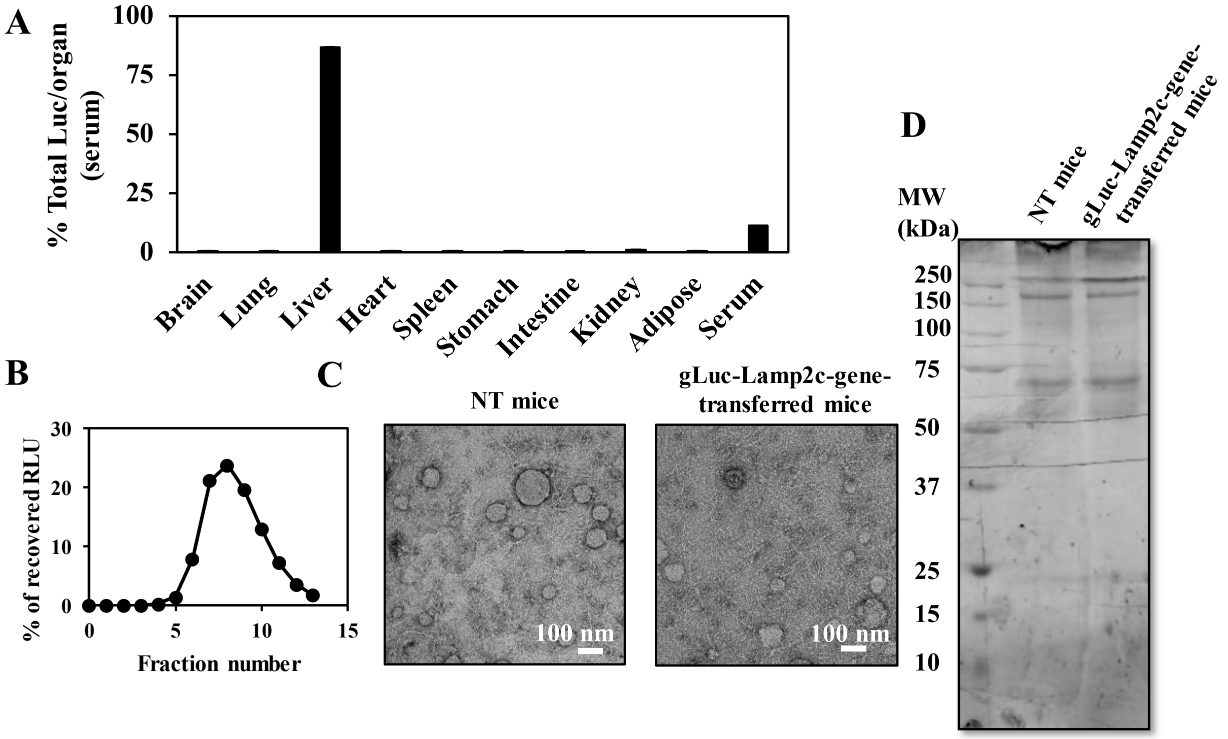

Fig. S6. sEV was isolated from the plasma of gLuc-Lamp2c gene-transferred mice and characterized, Related to Fig. 3. (A) Transgene expression of gLuc-Lamp2c in each organ or serum of gLuc-Lamp2c gene-transferred mice prepared by hydrodynamic injection of gLuc-Lamp2c pDNA. Results are shown as the percentage of gLuc activity of each organ or serum divided by the total recovered gLuc activity. (B) Typical SEC elution pattern of mouse serum from gLuc-Lamp2c gene-transferred mice. (C) TEM observation of sEV collected from NT-mice or gLuc-Lamp2c gene-transferred mice. Scale bar: 100 nm. (D) SDS-PAGE analysis of sEV collected from plasma of NT-mice or gLuc-Lamp2c transgenic mice.

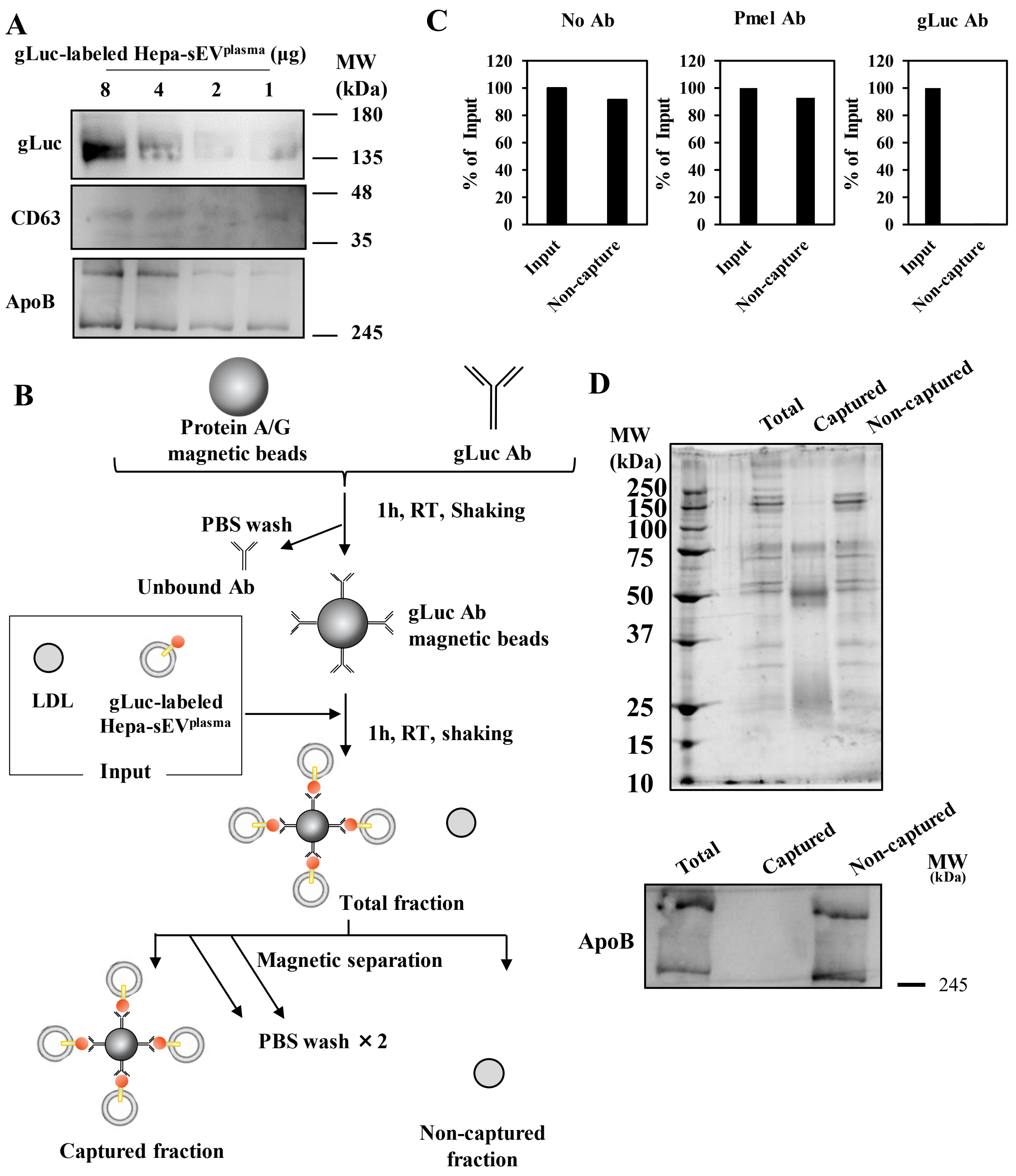

Fig. S7. gLuc-Lamp2c did not label LDL co-isolated in gLuc-labeled Hepa-sEVs^{plasma}, Related to Fig. 3. (A) Western blotting analysis of gLuc-labeled Hepa-sEVs^{plasma}-enriched SEC eluate. sEVs (1–8 μg) were loaded into SDS-PAGE and ApoB (LDL marker protein), CD63 and gLuc protein were detected by western blotting. (B) Schematic workflow for the immunocapture of of gLuc-labeled Hepa-sEV^{plasma} samples. (C) gLuc distribution after immunocapture using anti-Pme1 Ab (as a negative control Ab) or anti-gLuc Ab-coated magnetic beads. The results are shown as the percentage of gLuc activity of each fraction divided by the total recovered gLuc activity. (D) SDS-PAGE of total, captured, and non-captured fractions (upper). ApoB was detected by western blotting (lower).

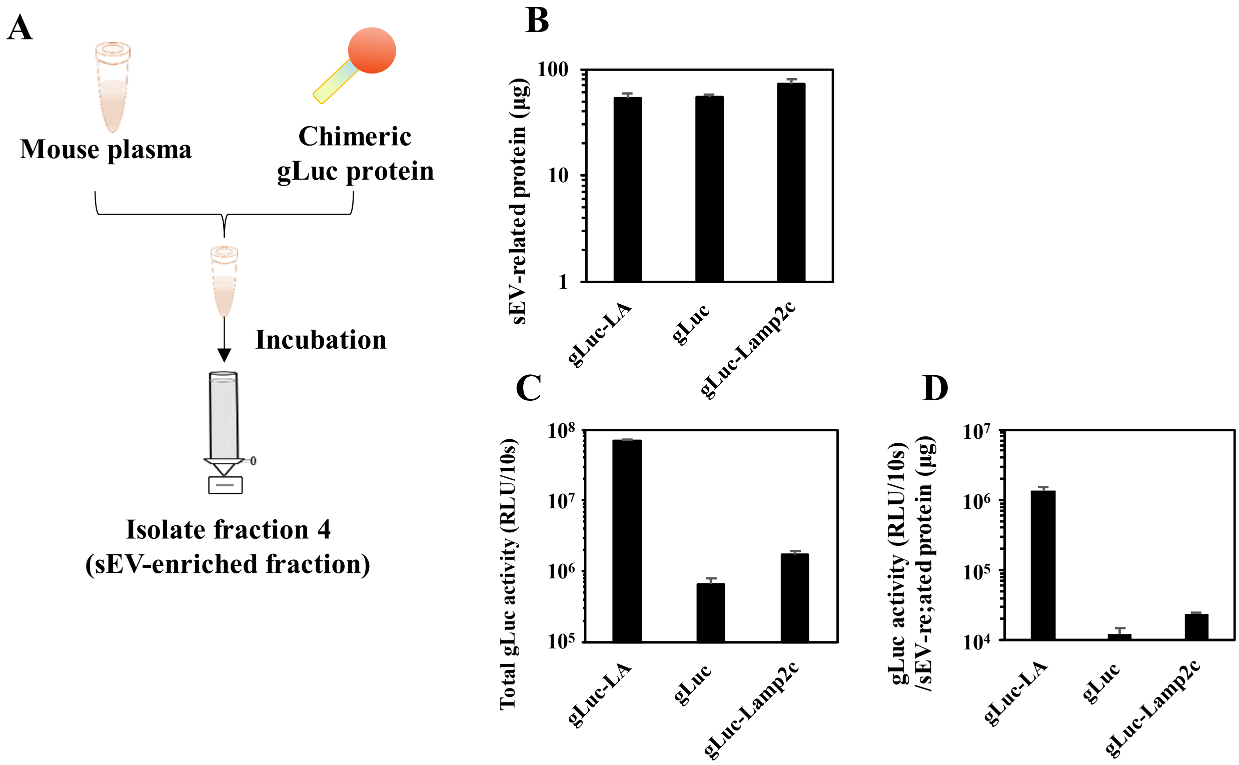

Fig. S8. Exogenous gLuc-Lamp2c hardly labeled to Plasma-sEVs, Related to Fig. 3. (A) Scheme of the exogenous labeling of gLuc-Lamp2c to Plasma-sEVs. Mouse plasma was incubated with gLuc-LA (as a positive control), gLuc (as a negative control), or gLuc-Lamp2c-enriched protein. After overnight incubation, the mixtures were subjected to SEC and the Plasma-sEV-enriched fraction was isolated. (B) The protein amount, and (C) the gLuc enzyme activity of the fraction was measured. (D) Based on the obtained results, the gLuc enzyme activity per protein amount was calculated. Results are shown as the average ± SD (n = 3).

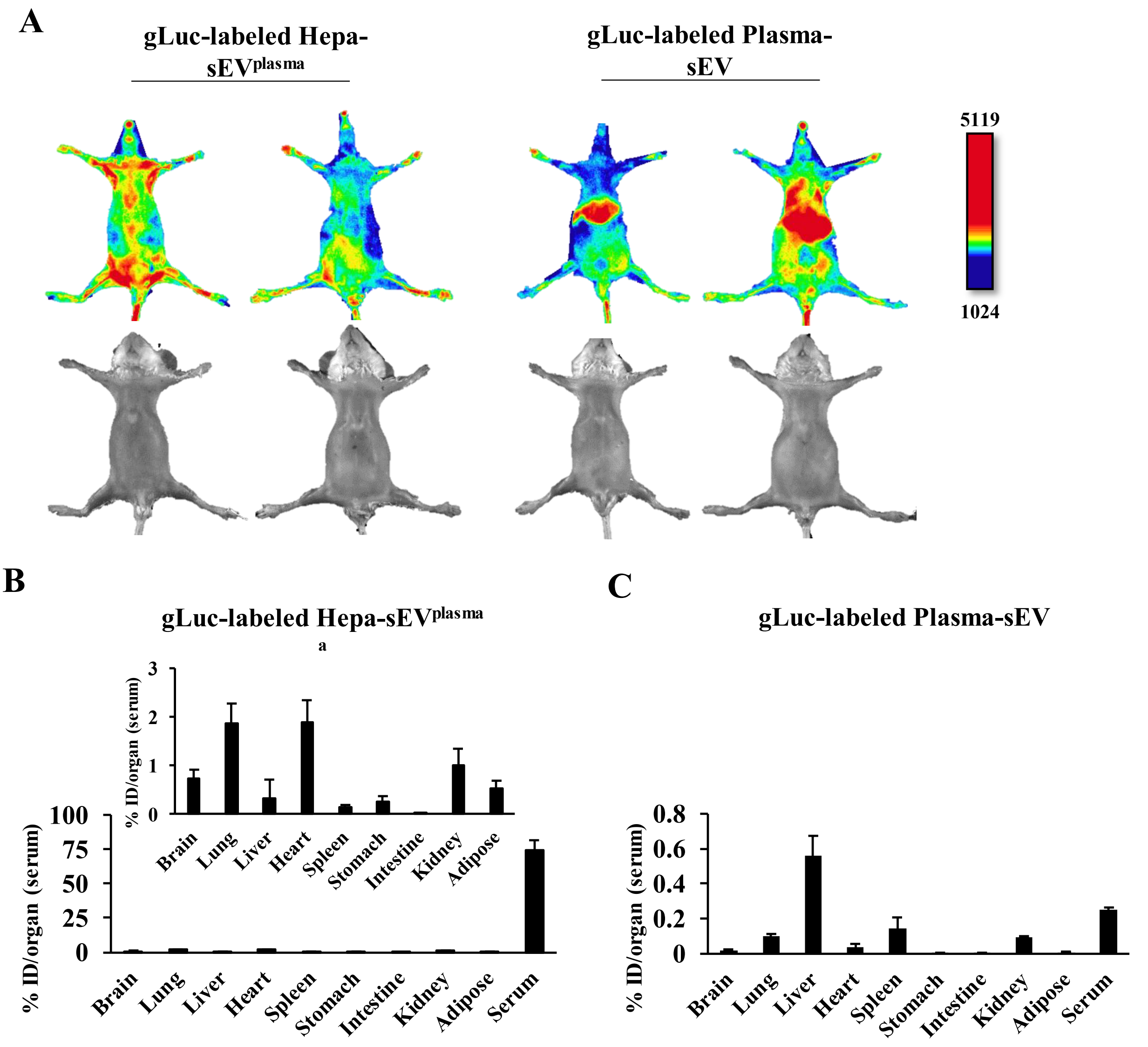

Fig. S9. gLuc-labeled Hepa-sEVs^{plasma} hardly distributed to liver compared to gLuc-labeled Plasma-sEVs after i.v. administration, Related to Fig. 4. (A) NT-mice were treated with gLuc-labeled Hepa-sEVs^{plasma} or gLuc-labeled Plasma-sEVs. The indicated sEVs were imaged 5 min after i.v. administration of the sEV samples through a bolus i.v. administration of coelenterazine (a gLuc substrate) and chemiluminescence was detected. (B), (C) Tissue distribution of gLuc activity 1 h after i.v. administration of (B) gLuc-labeled Hepa-sEV^{plasma} or (C) gLuc-labeled Plasma-sEV into NT-mice. The results are expressed as mean ± SD (n = 3).

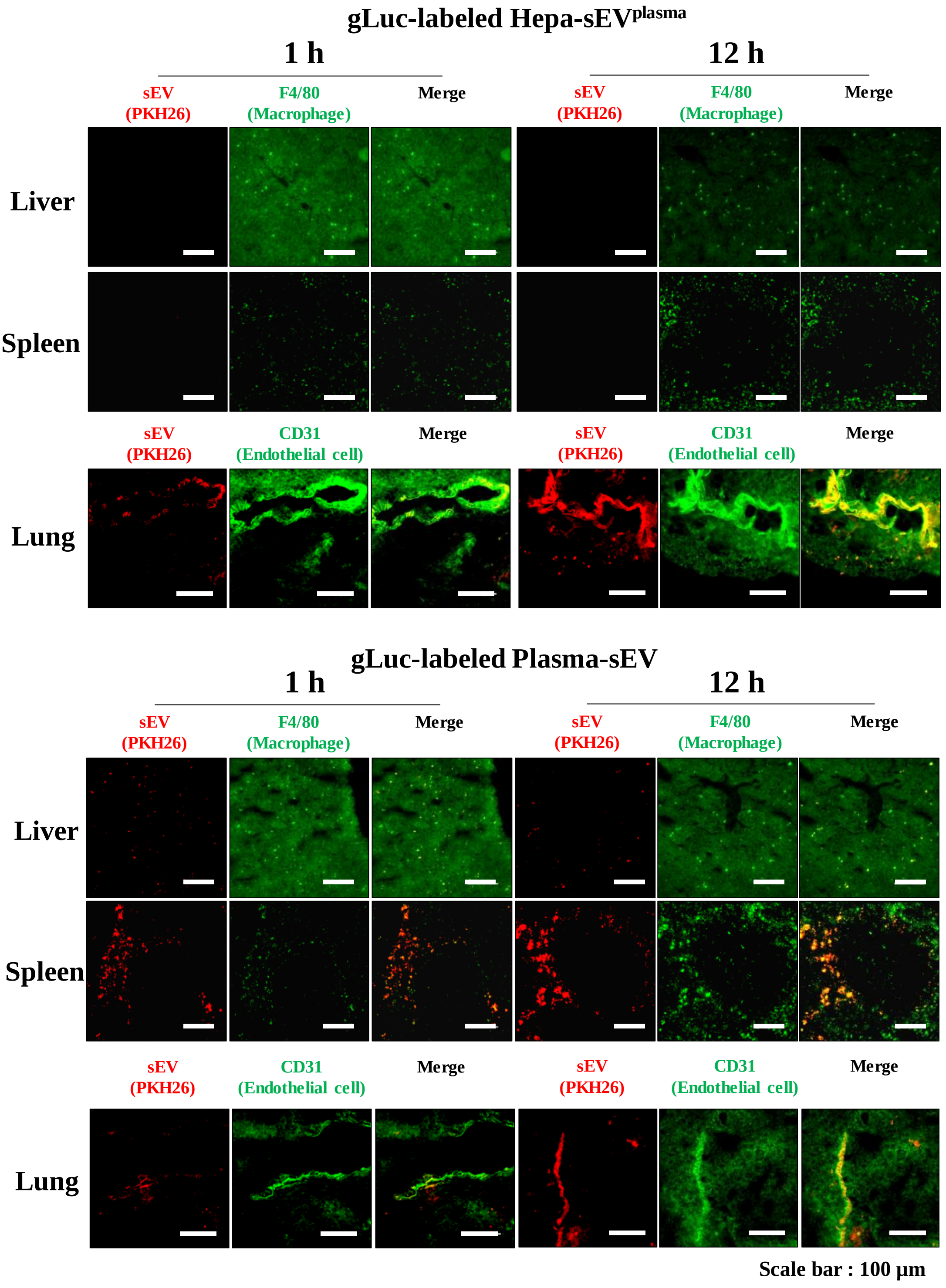

Fig. S10. gLuc-labeled Hepa-sEVs^{plasma} distributed in the lung, but not in the liver and spleen, Related to Fig. 4. The indicated sEVs labeled with PKH26 were admininstered into mice by i.v. administration (5 µg/dose). After 1 or 12 h of injection, the liver, spleen, or lung were collected and cut into a cryostat section. The section was stained with F4/80-specific Ab (liver or spleen) or CD31-specific Ab (lung) and observed by fluorescence microscopy. Scale bar: 100 µm.

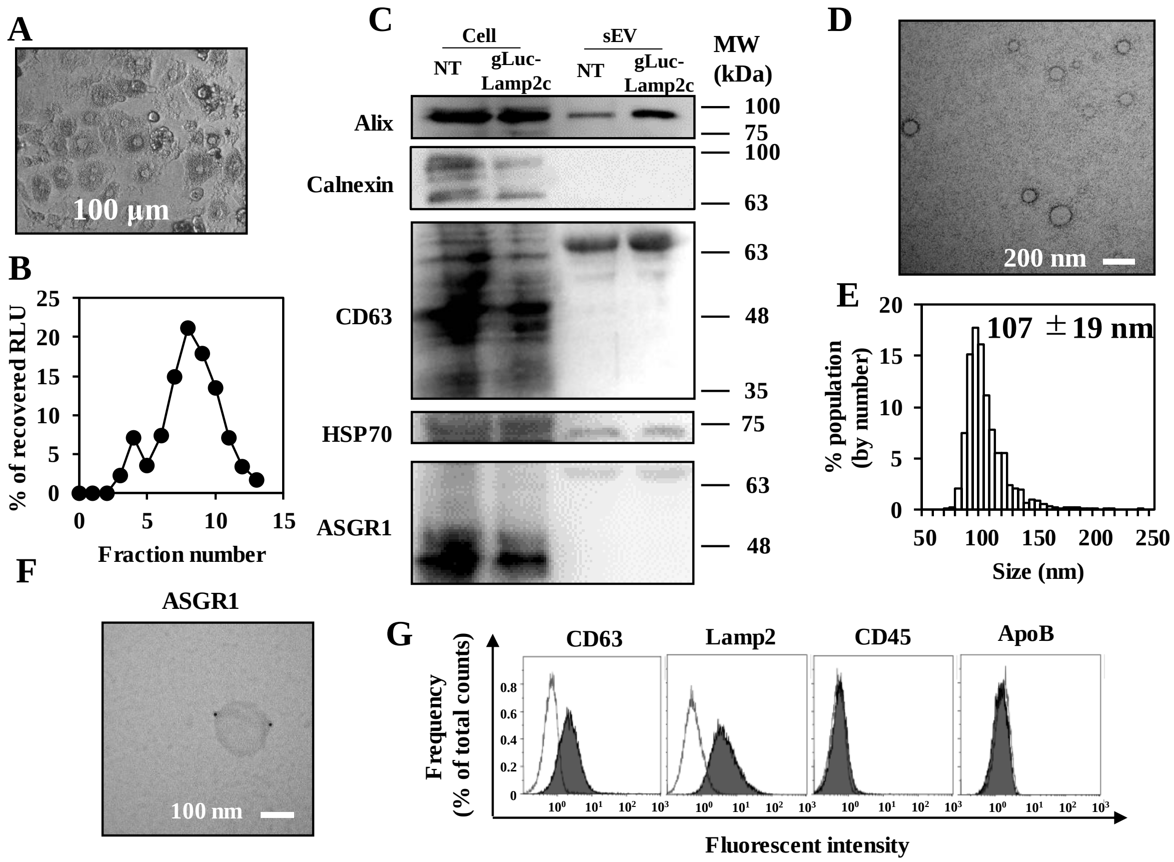

Fig. S11. gLuc-labeled Hepa-sEV from cultured hepatocytes was isolated and characterized, Related to Fig. 5. (A) Microscopic observation of primary hepatocytes isolated from gLuc-Lamp2c transgene mice. Scale bar: 100 µm. (B) Typical elution pattern of the culture medium subjected to SEC. Results are shown as the percentage of gLuc activity of each fraction divided by the total recovered gLuc activity. (C) Western blotting analysis of sEV marker proteins (CD63, Alix, and HSP70), a hepatocyte marker protein (ASGR1) as well as a negative sEV marker protein (Calnexin) in Hepa-sEVs or gLuc-Lamp2c-labeled Hepa-sEVs. (D)-(G) gLuc-Lamp2c-labeled Hepa-sEV from cultured hepatocyte sample was immunocaptured by gLuc Ab-coated magnetic beads. The sEVs in the eluate were observed by (D) TEM (scale bar: 200 nm) and analyzed by (E) the qNano instrument for the size measurement. (F) sEVs in the eluate were stained with protein A-gold nanoparticles after reacting with an anti-ASGR1 Ab (right), followed by TEM observation. Scale bar: 100 nm. (G) The sEVs-bead complexes were subsequently stained with the indicated Ab and analyzed by flow cytometry. BSA was set as a control sample against sEV.

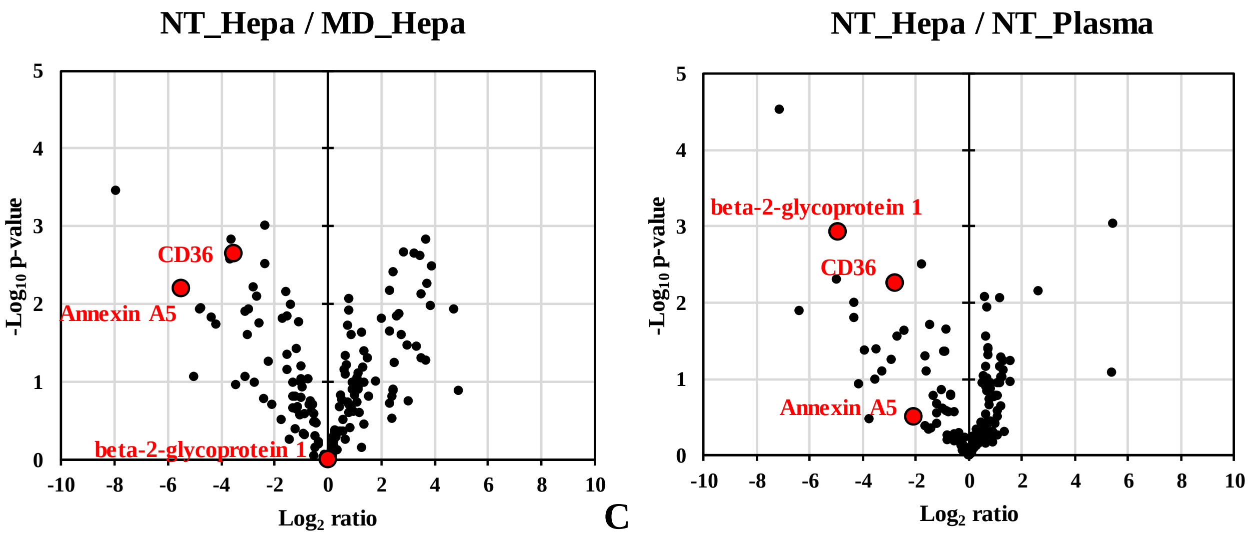

Fig. S12. Protein composition of gLuc-labeled Hepa-sEVs^{plasma} from non-treated mice, gLuc-labeled Plasma-sEVs from NT mice and gLuc-labeled Hepa-sEVs^{plasma} from MD mice compared by unbiased proteomics, Related to Fig. 5 (A) gLuc-labeled Hepa-sEV^{plasma} from NT-mice (NT_Hepa), gLuc-labeled Hepa-sEV^{plasma} from MD-mice (MD_Hepa) and gLuc-labeled Plasma-sEV from NT-mice (NT_plasma) were concentrated through immunocapture by gLuc Ab-coated magnetic beads, followed by elution. Thereafter, proteome analysis of the sEV samples was performed. The 154 identified proteins were ranked in volcano plots according to their statistical P-value (y-axis) and their relative abundance ratios (log$_2$ fold-change, x-axis) between NT_Hepa versus MD_Hepa (left) and NT_Hepa versus NT_Plasma (right).

Table S1.

PS$^{(-)}$ sEVs derived from B16 cells showed slower clearance from blood circulation than gLuc-Lamp2c-labeled bulk sEVs derived from B16 cells after i.v. administration into NT-mice, Related to Fig. 1.

Sample	Mice (*N*)	A	α	B	β	t1/2α (min)	t1/2β (min)	AUC (% ID·h/mL)	CL (mL/h)	MRT (h)
**Bulk sEV**	NT (*N*=9)	6.07± 0.82	0.210± 0.037	1.32± 0.18	0.00386± 0.00110	6.06± 2.25	564± 341	3.74± 0.31	28.5± 2.4	1.44± 0.03
**PS$^{(-)}$-sEV**	NT (*N*=6)	28..7± 6.5	0.0378± 0.0064	41.2± 10.5	0.0018± 0.0004	23.1± 4.8	693± 248	142± 33	1.03± 0.24	1.67± 0.02

Table S2.

GLuc-labeled Hepa-sEVs^{plasma} showed slower clearance from blood circulation than gLuc-labeld Plasma-sEVs after i.v. administration into NT-mice, Related to Fig. 3.

Sample	Mice (*N*)	A	α	B	β	t1/2α (min)	t1/2β (min)	AUC (% ID·h/mL)	CL (mL/h)	MRT (h)
**gLuc-labeled Hepa^{plasma}-sEV**	NT (*N=15*)	17.3± 7.7	0.0049± 0.0007	48.4± 8.1	0.00151± 0.00021	205± 33	1315± 675	211± 15	0.51± 0.03	1.82± 0.02
**gLuc-labeled Plasma-sEV**	NT (*N=6*)	8.43± 0.88	0.190± 0.046	0.253± 0.040	0.0104± 0.0014	5.46± 1.27	74.6± 10.6	1.07± 0.20	117± 22	0.419± 0.05

Table S3.

Pharmacokinetic (PK) parameters of gLuc-labeled Hepa-sEVs^{plasma} collected from NT-mice showed slower clearance from blood circulation than those collected from MD-mice after i.v. administration into NT-mice, Related to Fig. 5.

Origins of gLuc-labeled Hepa-sEV^{Plasma}	Mice (*N*)	A	α	B	β	t1/2α (min)	t1/2β (min)	AUC (%ID·h/mL)	CL (mL/h)	MRT (h)
**NT mice**	NT (*N=15*)	17.3± 7.7	0.0049± 0.0007	48.4± 8.1	0.00151± 0.00021	205± 33	1315± 675	211± 15	0.51± 0.03	1.82± 0.02
**MD mice**	NT (*N=3*)	1.54± 1.25	0.0480± 0.0110	6.63± 0.66	0.00337± 0.00010	16.5± 3.0	206± 6	17.8± 1.16	5.69± 0.40	1.64± 0.02
